# Promoting physical activity in rheumatoid arthritis through a physiotherapist led behaviour change-based intervention (PIPPRA): a feasibility randomised trial

**DOI:** 10.1007/s00296-024-05544-1

**Published:** 2024-03-04

**Authors:** Louise Larkin, Seán McKenna, Tadhg Pyne, Paul Comerford, Anusha Moses, Ailish Folan, Stephen Gallagher, Liam Glynn, Alexander Fraser, Bente Appel Esbensen, Norelee Kennedy

**Affiliations:** 1 Faculty of Education and Health Sciences, School of Allied Health, Discipline of Physiotherapy, University of Limerick, Limerick, Ireland; 2 Health Research Institute, University of Limerick, Limerick, Ireland; 3 Department of Psychology, Faculty of Education and Health Sciences, University of Limerick, Limerick, Ireland; 4 School of Medicine, Faculty of Education and Health Sciences, University of Limerick, Limerick, Ireland; 5 Department of Rheumatology, University Limerick Hospitals Group, Limerick, Ireland; 6 Copenhagen Center for Arthritis Research, Center for Rheumatology and Spine Diseases, Rigshospitalet, Glostrup, Denmark; 7 Department of Clinical Medicine, Faculty of Health and Medical Sciences, University of Copenhagen, Copenhagen, Denmark

**Keywords:** Rheumatoid arthritis, Exercise, Behaviour change, Physical activity, Physiotherapy, Feasibility study

## Abstract

Physical activity (PA) is recommended as a key component in the management of people with rheumatoid arthritis (RA). The objective of this study was to examine the feasibility of a physiotherapist led, behaviour change (BC) theory-informed, intervention to promote PA in people with RA who have low levels of current PA. A feasibility randomised trial (ClinicalTrials.gov NCT03644160) of people with RA over 18 years recruited from outpatient rheumatology clinics and classified as insufficiently physically active using the Godin−Shephard Leisure Time Physical Activity Questionnaire. Participants were randomised to intervention group (4 BC physiotherapy sessions in 8 weeks) delivered in person/virtually or control group (PA information leaflet only). Feasibility targets (eligibility, recruitment, and refusal), protocol adherence and acceptability were measured. Health care professionals (HCPs) involved in the study and patients in the intervention and control arms were interviewed to determine acceptability. Descriptive statistics were used to analyse the data with SPSS (v27) with interviews analysed using content analysis using NVivo (v14). Three hundred and twenty participants were identified as potentially eligible, with *n* = 183 (57%) eligible to participate, of which *n* = 58 (32%) consented to participate. The recruitment rate was 6.4 per month. Due to the impact of COVID-19 on the study, recruitment took place over two separate phases in 2020 and 2021. Of the 25 participants completing the full study, 23 were female (mean age 60 years (SD 11.5)), with *n* = 11 allocated to intervention group and *n* = 14 to control. Intervention group participants completed 100% of sessions 1 & 2, 88% of session 3 and 81% of session 4. The study design and intervention were acceptable overall to participants, with enhancements suggested. The PIPPRA study to improve promote physical activity in people with RA who have low PA levels was feasible, acceptable and safe. Despite the impact of COVID-19 on the recruitment and retention of patients, the study provides preliminary evidence that this physiotherapist led BC intervention is feasible and a full definitive intervention should be undertaken. Health care professionals involved in the study delivery and the patient participants described a number of positive aspects to the study with some suggestions to enhance the design. These findings hence inform the design of a future efficacy-focused clinical trial.

**Supplementary Information:**

The online version contains supplementary material available at 10.1007/s00296-024-05544-1.

## Background

Rheumatoid arthritis (RA) is a chronic autoimmune condition mainly affecting the joints and can lead to a 1.5–1.6 fold higher mortality rate than in the general population [1]. Prevalence of RA is varied with an average point and period prevalence of 51 in 10,000 and 56 in 10,00 reported in a review of studies from 1986 to 2014 [2].

Recommended management of RA includes pharmacological and non-pharmacological treatments with physical activity (PA) being an important component in the non-pharmacological management of RA [3, 4]. However, people with RA tend to have low PA levels [5, 6] thus improving PA levels in this group is an important part of treatment.

To date, interventions targeting longer term PA change in people with RA have had some success [7, 8], with challenges in improving healthy PA (> or = 30 min, moderately intense activity, most days of the week) [7] and transitioning to and maintaining more intense levels of PA [8]. Adopting a behaviour change approach to PA promotion is a key aspect of better intervention design [9, 10]. Adopting the Behaviour Change Wheel [11] can guide the determination of the factors to be addressed in a BC intervention to promote PA. Health professional involvement with people with RA in promoting PA is one important aspect of intervention delivery [12]. In addition, targeting participants with the existing low levels of PA should be considered as low PA levels have been shown to be an independent risk factor for number of hospital admissions and duration of hospitalisation in people who have RA [13]. Therefore, increasing and maintaining PA levels in people with RA may serve to reduce healthcare costs and enhance the health outcomes of the RA population [14].

Guided by the Medical Research Council framework for complex interventions [15] the authors undertook extensive systematic literature reviews [9, 10], objective PA measurement validation [16] and qualitative interviews with people with RA [17] and with rheumatology health professionals [18] to design a robust intervention [19] to address the issues above. Following the completion of these underpinning studies, a novel BC physiotherapist led intervention for adults with RA to promote physical activity (PIPPRA study) was developed and a feasibility study was designed. The aim of this study was to explore the overall feasibility of PIPRRA with the following objectives: (1) to determine the number of eligible participants, the recruitment numbers and rate, protocol adherence; (2) to evaluate the acceptability of PIPPRA in both patients and HCPs involved in recruitment and delivery of the intervention and (3) to undertake exploratory analyses of the outcome data to develop a power calculation for a future trial.

## Methods

### Study design

The study design was a feasibility randomised trial with the published protocol (version 1) [19] and the final trial protocol registered on ClinicalTrials.gov NCT03644160. Ethical approval was granted by the University Hospital Limerick (UHL) Research Ethics Board (approval number 064/19). Reporting followed the relevant extensions of the Consolidated Standards of Reporting Trials [20].

### Participants and setting

Inclusion criteria for the intervention part of the study were: adults (over 18 years) with a diagnosis of RA based on the American College Rheumatology 2010 criteria and low PA levels using the Godin−Shephard Leisure−Time Physical Activity Questionnaire (GSLPAQ) [21]. Patients identified from clinic charts as having a diagnosis of RA were approached by the study research assistant who introduced the study and completed the GSLPA) with each patient. If the patient had a score of less than 14 on the GSLPAQ they were given an information leaflet on the study and any questions were answered. A consent form was given and a time was arranged for a follow-up call within the next week to determine the person’s interest or not in participating the in the study. Informed consent was then obtained prior to formal study enrolment at the arranged baseline meeting with the study team. Patients continued with their usual care throughout the study.

For the interview part of the study patients in both intervention and control arms were invited to participate following completion of the main study. Health care professionals involved in rheumatology clinics the patients were recruited from (rheumatologists, medical teams and nursing staff) as well as the sessional physiotherapists who delivered the intervention were invited to participate on completion of the study. Informed written consent was recorded for each participant in the interview part of the study.

The study was conducted at University Hospital Limerick (UHL), Ireland in the outpatient rheumatology department (recruitment) and the hospital’s Clinical Education and Research Centre (CERC) (intervention delivery and assessments). Participants were recruited from weekly outpatient rheumatology clinics in UHL across two periods (due to interruption from Covid-19) between October 2019 and March 2020 and November 2020 to May 2021 (9 months total).

Patient safety was monitored throughout the study by the study team. Discontinuation criteria were determined at the outset of the study as follows: a) immediate discontinuation will occur, regardless of the stage of the study, if participants develop any severe adverse events during the study which may or may not be related to the intervention—such as chest pain; b) Participants could also choose to discontinue the intervention at any time for any reason. The type and frequency of adverse events were recorded and reported to the study Data and Safety Monitoring Committee (DSMC) if they occurred.

### Randomisation, allocation concealment and blinding

The CERC Unit provided a computer randomisation system. Randomisation was performed by computer generated random numbers with a 1:1 allocation ratio to the intervention or control arms. The allocation sequence was generated independent of the study team. Allocations were stored in a locked cabinet. Participants were informed of allocation by the research assistant after completion of their baseline assessments. Each participant was assigned a code number, which was used on all outcome assessments in place of their names. The assessor was blinded to group allocation. The person assigning the allocations was blinded to allocation until after the participant was assigned. The statistician who completed the data analysis was blinded to group allocation.

### Intervention and control group

The PIPPRA trial is a theoretically underpinned physiotherapist led BC intervention to promote PA delivered over 4 1:1 sessions by a sessional physiotherapist in person or virtually over an 8 week period. The intervention was tailored to each individual depending on the discussion with the sessional physiotherapist on the type of PA they were undertaking. Each session was a maximum of one hour. The original design was in-person intervention delivery only; however due to the timing of the study during the Covid-19 pandemic, virtual sessions were necessitated to ensure the completion of the study. Virtual sessions were delivered using WhatsApp video calls. The in-person sessions were delivered in a meeting room with minimal infrastructure and no exercise equipment. This was deliberate to ensure the transfer of the intervention delivery to any setting and eliminate the reliance on specialist settings or equipment.

PIPPRA comprises individual sessions based on the specific BC techniques, as described by the Behaviour Change Taxonomy [11]. A detailed description of the final programme is included in supplementary file 1 and in the protocol paper [19]. Briefly, across the 4 sessions participants were guided using a range of ‘education’ techniques’ (e.g. instruction on how to perform a behaviour, discussion on health consequences), ‘enablement’ techniques (e.g. goal setting, problem solving, action planning, feedback, self-monitoring, social comparison) and ‘modelling’ techniques. A PA behaviour plan was also prepared by each participant and reviewed at each session and adjusted as needed and agreed by the participant and therapist. Both control and intervention group received an information booklet about PA based on the existing guidelines for people with RA (Supplementary file 2).

### Therapist intervention training

The sessional physiotherapist was trained in BC techniques and motivational interviewing by the study’s postdoctoral researcher and co-author (LL). The postdoctoral researcher was a qualified physiotherapist with a PhD in the area of behaviour change for PA in rheumatology and also co-applicant on the study and lead author on many of the underpinning studies. The sessional physiotherapists were newly qualified therapists with no experience in specialist rheumatology settings. The rationale for involving newly qualified therapists was to explore if a BC intervention could be delivered with minimal specialist training. A detailed session log of each session was maintained by the sessional physiotherapists (supplementary file 3a and 3b).

### Modifications to study design due to COVID-19

COVID-19 restrictions in Ireland came into effect in March 2020 and necessitated a transition, after a 5 month pause of the study, from in-person to remote, virtual assessments and intervention delivery [22]. The COVID-19 pandemic also resulted in lost data as we were unable to follow-up some participants who withdrew from the study for reasons linked to Covid-19.

### Data collection

The data collection took place at baseline (Time 0 (T0), 12 weeks (Time 1 (T1)) and 24 weeks (Time 2 (T2)). Questionnaires were completed via phone call with participants ActivPal devices were sent to and returned via post. The data were downloaded from the ActivPal to the study laptop and the output file was uploaded to the e-Case report form database (Clindox Limited, hosted by the University of Limerick encrypted server). Participant’s out of pocket expenses were reimbursed using a shopping voucher.

### Primary outcomes

A number of feasibility targets were calculated:

#### Recruitment and retention

- Feasible eligibility—the total number of eligible participants from UHL group rheumatology clinics
- Recruitment rate—the number (%) of participants recruited and the rate of participants recruited to the study.
- Refusal numbers—the number (%) of eligible participants that refused to participate and reasons why.

#### Protocol adherence

- Minimum average attendance—the number of participants who attended over 80% of the intervention sessions.
- Minimum outcome assessment target—retention of at least 80% of recruited participants with valid 12 (T1) and 24 (T2)—week primary outcome data, i.e. less than 20% attrition at outcome assessments at 12 and 24-week follow-up

#### Intervention acceptability

Semi-structured interviews were undertaken on completion of recruitment and the intervention with patients and the HCPs (rheumatology clinic medical team and nursing staff and sessional physiotherapists). Patients and HCPs were provided with verbal and written information about the study. Each participant provided informed consent prior to participation. The interviews aimed to determine patient and HCP acceptability of the intervention, understand some of the trial design and processes and to consider the acceptability of the secondary outcomes used. Interview questions for the interview guide (Supplementary file 4) were developed from an extensive literature review on behaviour change interventions to promote physical activity behaviour in people who have RA [9, 10] and from the previous qualitative research in this area [17, 18]. Interviews were audio-recorded and conducted by co-authors LL and S McK, chartered physiotherapists and researchers who have experience of undertaking and analysing qualitative interviews. Audio recordings from the semi-structured interviews were transcribed, anonymised and saved to a password protected laptop from University of Limerick. Each interviewee was offered a copy of their transcript to review and advised to amend the transcript if necessary. Once interviewees were satisfied that the transcript reflected their views and opinions accurately the finalised transcript was included in data analysis. Preliminary data analysis was conducted concurrently with data collection, to enhance understanding about the questions being asked and facilitated minor revisions of the questions. The analytical approach for this data was content analysis. NVivo (version 14 QSR International) was used to support the qualitative analysis.

### Secondary outcomes

#### Physical activity

The ActivPAL™ (PAL Technologies Ltd, Glasgow, UK) measured time spent sitting, standing, lying and time spent in moderate and vigorous intensity aerobic physical activity (MVPA) [16]. It has been validated in RA samples [23]. The ActivPAL™ is a small, lightweight activity monitor that uses proprietary algorithms. It is a tri-axial accelerometer that produces a signal related to thigh inclination and needs no calibration before use. Recordings were processed for daily minutes of moderate to vigorous PA. The ActivPAL™ was worn for 8 days beginning week 1 before the start of intervention (T0, for 8 days 1-week post-intervention (T1) and at the 24-week assessment (T2). The first 24 h of recording were not included in the analysis to minimise the effects of reactivity. A minimum recording duration of 3 days from the 7-day period, including at least one weekend day was required for data processing; samples of lower than 3 days were not included.

The Yale Physical Activity Survey (YPAS) [24] measured participants self-report PA and has been used in the previous arthritis studies and older adults [25, 26]. The 2-part YPAS measures PA over a time period of a typical recent week (part 1) and from the past month (part 2). The total energy expenditure per week was calculated from part 1 of the questionnaire for each participant at each time point.

#### Psychological beliefs

The theory of Planned Behaviour Questionnaire (TPBQ) consists of three elements; beliefs about the likely consequences of the behaviour (behavioural beliefs), beliefs about the normative expectations of others (normative beliefs), and beliefs about the presence of factors that may facilitate or impede performance of the behaviour (control beliefs) [27]. Behavioural beliefs produce a favourable or unfavourable attitude toward the behaviour; normative beliefs result in perceived social pressure or subjective norm; and control beliefs give rise to perceived behavioural control. Thus, attitude toward the behaviour, subjective norm, and perception of behavioural control led to the formation of a behavioural intention. The TPBQ aims to capture all three elements which contribute to behavioural intention.

#### Disease activity

The Disease Activity Score 28 (DAS28) [28] was used to combine single measures into an overall, continuous measure of RA disease activity. The DAS28 includes a 28 tender joint count, a 28 swollen joint count, erythrocyte sedimentation rate, and a general health assessment on a visual analogue scale. After study interruption due to COVID-19 this measure was not recorded as participants were not able to attend the unit physically for blood draws.

#### Pain

Pain was recorded using a 10 cm visual analogue scale (VAS), which is sensitive to detecting changes in pain in inflammatory conditions and has good reliability and validity [29].

#### Fatigue

The Bristol Rheumatoid Arthritis Fatigue Multi-Dimensional Questionnaire (BRAF-MDQ) [30] measured the impact of fatigue for people with RA and disease specific. It has acceptable to good convergent validity [31] and consists of 20 items combined to create 5 scores, where a higher score reflects worse fatigue. The total score range is 0–70.

#### Sleep

Sleep was assessed using the Pittsburgh Sleep Quality Index (PSQI) [32] and has been used in previous arthritis studies [33, 34]. It is a 19-item self-rated questionnaire for evaluating subjective sleep quality over the previous month. The 19 questions are combined into 7 clinically-derived component scores, each weighted equally from 0 to 3. The clinical and psychometric properties of the PSQI have been formally evaluated by several research groups [33].

#### Quality of life

The Rheumatoid Arthritis Quality of Life Questionnaire (RAQoL) measured disease related quality of life [35] on ADLs, social interaction, emotional well-being, and relationships. The questionnaire consists of 30 statements that have a yes/no response. Items are scored one for yes and zero for no. Scores for each item are summed to give an overall quality of life score with a higher score indicating a poorer quality of life.

#### Sample size and data analysis

The target sample size for the pilot study of 40 participants, with 20 participants in both the control and intervention groups was determined pragmatically. This sample size was expected to provide sufficient data to meet the primary outcomes and was in the appropriate range for pilot studies [36]. Descriptive statistics were used to determine the recruitment and retentions rates and for the secondary outcomes. Data analysis was undertaken in SPSS version 27 (IBM Corporation, New York, USA). Given the small sample size it was not deemed appropriate to present differences between arms.

#### Progression criteria

The following criteria were agreed for progression to a full trial.

1. In order for a definitive trial to be feasible we project that we need to recruit participants at a minimum recruitment rate of 4 per month; i.e. 48 per year. The primary feasibility target is a minimum recruitment rate of 4 participants per month.
2. Following recruitment of participants, a minimum attendance at 100% of the intervention sessions is expected. A second feasibility target is thus a minimum average attendance by recruited participants of 100% of the intervention sessions.
3. Outcome assessment at 12-week follow-up—retention of at least 80% of recruited participants with valid outcome assessments, i.e. less than 20% attrition. Thus a third feasibility target is a minimum retention of 80% of recruited participants providing valid 12-week outcome data

## Results

**Fig. 1:**
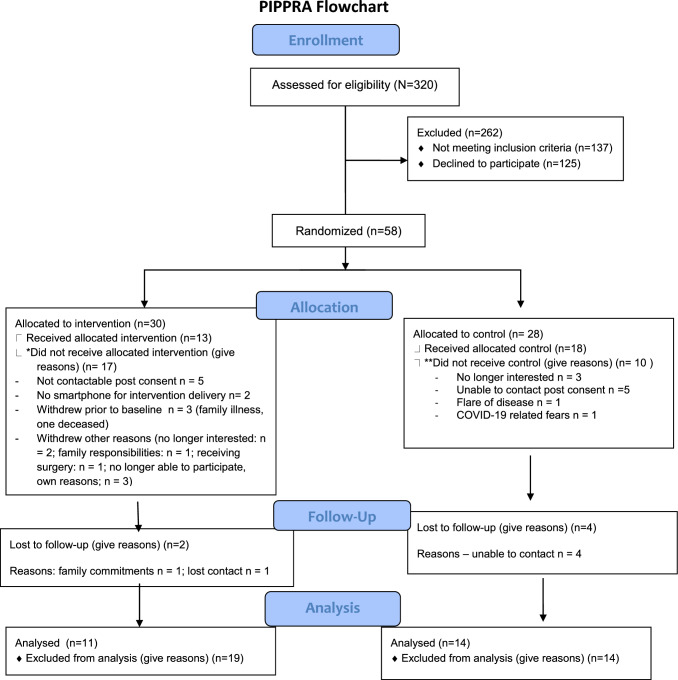
Demographic profile of patient participants

Recruitment occurred over two separate time periods between October 2019 and March 2020 and November 2020 to May 2021 (9 months total). A total of 320 participants were identified at the outpatient clinics as potentially eligible. Baseline demographic characteristics are reported in Table 1. The flow of participants through the study is shown in Fig. 1.

**Table 1:** Patient demographics

	Intervention group (*n* = 11)	Control group (*n* = 14)	Total(*n* = 25)
	Mean (sd)	Mean (sd)	Mean (sd)
Age	61.0 (11.6)	58.7 (11.8)	60.0 (11.5)
*Gender*			
Male	1 (9.1%)	1 (7.1%)	2 (8.0%)
Female	10 (90.9%)	13 (92.9%)	23 (92.0%)
Disease duration	13.5 years (12.8)	13.3 years (8.2)	13.4 years (10.3)
*Marital status*			
Cohabiting	1 (9.1%)	0 (0.0%)	1 (4.0%)
Married	10 (90.9)	11 (78.6%)	21 (84.0%)
Separated/divorced	0 (0.0%)	1 (7.1%)	1 (4.0%)
Single	0 (0.0%)	2 (14.3%)	2 (8.0%)
*Employment*			
Employed	4 (36.4%)	3 (21.4%)	7 (28.0%)
Unemployed	7 (63.6%)	11 (78.6%)	18 (72.0%)
*Education*			
Primary level	1 (9.1%)	1 (7.1%)	2 (8.0%)
Second level	6 (54.5%)	7 (50.0%)	13 (52.0%)
Third level	4 (36.4%)	6 (42.9%)	10 (40.0%)

### Primary outcome measures

#### Recruitment and retention

Of the total assessed for eligibility (*n* = 320), *n* = 262 were excluded with 137 of these not meeting the inclusion criteria and 125 declining the invitation to participate. The remaining eligible participants (*n* = 58 (55%) consented to participate with a recruitment rate of 6.4 per month. Owing to impact of COVID-19 on the study *n* = 25 (43%) participants completed the study (*n* = 11 (44%) in intervention and *n* = 14 (56%) in control). Of the 25, *n* = 23 (92%) were female, the mean age was 60 years (SD 11.5). Further details of the demographic profile of the participants is shown in Table 1. No adverse events were reported.

#### Protocol adherence

Intervention group participants completed 100% of session 1 and 2, 88% session 3 and 81% session 4. Participants reported no adverse events (AEs) during the 24-week intervention period. The percentage of participants who completed assessments at T1 was 53% and 69% at T2.

#### Secondary outcome measures

Descriptive statistics for secondary outcomes are reported in Table 2. In line with the objectives of the study the inclusion of outcomes was to provide preliminary evidence of the efficacy of the intervention and estimation of the population standard deviation for objective PA to inform sample size calculations for a future definitive trial. No within group or between group analyses of differences was undertaken as this was not the aim of the study.

**Table 2:** Descriptive statistics for secondary outcomes

Outcomes and instruments	Intervention			Control		
	Baseline	*Time 1	**Time 2	Baseline	*Time 1	**Time 2
*Physical activity – self report (YPAS*^*1*^*)*						
Mean (SD)	146.27 (193.73)	120.88 (70.79)	132.00 (70.65)	91.75 (85.86)	58.03 (50.61)	124.31 (69.75)
95% CI	16.12; 276.42	73.32; 168.45	84.54; 179.47	42.18; 141.32	28.81; 87.25	84.04; 164.58
*Physical activity – objective*^*2*^* (step count)*						
Mean (SD)	32,616.91 (11,415.85)	23,410.91 (16,559,11)	22,740.00 (13,058.76)	28,299.28 (14,625.03)	24,067.00 (26,865.05)	31,839.57 (19,298.93)
95% CI	24,947.63; 40,286.18	12,286.34; 34,535.47	13,967.00; 31,512.99	19,855.05; 36,743.53	8,555.58; 39,578.41	20,696.70; 42,982.44
*Pain (VAS*^*3*^*)*						
Mean (SD)	4.18 (2.40)	4.90 (1.91)	4.50 (1.90)	3.71 (2.55)	5.10 (2.77)	4.79 (2.19)
95% CI	2.57; 5.80	3.53; 6.27	3.14; 5.86	2.24; 5.19	3.12; 7.08	3.52; 6.05
*Disability (HAQDI*^*4*^*)*						
Mean (SD)	0.85 (0.57)	0.94 (0.57)	1.03 (0.65)	0.71 (0.56)	0.71 (0.52)	1.52 (0.69)
95% CI	0.47; 1.23	0.54; 1.35	0.56; 1.49	0.38; 1.03	0.34; 1.09	1.12; 1.92
*Fatigue (BRAF MDQ*^*5*^*)*						
Mean (SD)	18.18 (8.98)	14.82 (13.15)	18.55 (11.84)	15.71 (12.29)	13.93 (15.27)	20.21 (10.85)
95% CI	12.15; 24.21	5.98; 23.65	10.59; 26.50	8.62; 22.81	5.11; 22.74	13.95; 26.48
*Psychological constructs (TPB*^*6*^*)*						
Mean (SD)	22.00 (4.84)	19.91 (7.76)	20.27 (8.63)	21.79 (5.67)	15.50 (11.43)	25.36 (6.86)
95% CI	18.75; 25.25	14.69; 25.13	14.48; 26.07	18.51; 25.06	8.90; 22.10	21.40; 29.32
*Quality of life (RA QoL*^*7*^*)*						
Mean (SD)	15.18 (6.08)	14.73 (7.23)	17.09 (7.33)	18.43 (5.06)	11.50 (10.02)	14.86 (6.47)
95% CI	11.10; 19.27	9.87; 19.58	12.17; 22.01	15.50; 21.35	5.71; 17.29	11.12; 18.59
*Sleep (PSQI*^*8*^*)*						
Mean (SD)	10.2 (3.61)	10.1 (2.74)	11 (3.16)	10.1 (3.63)	9.88 (3.14)	11 (2.68)
95% CI	7.61; 12.78	7.83; 12.42	5.97; 16.03	7.88; 12.27	7.47; 12.30	8.18; 13.81

*Time 1–12 weeks

**Time 2–24 weeks

^1^YPAS – Yale Physical Activity Scale – total energy expenditure per week

^2^Objective PA measured with ActivPAL™ accelerometer – step count

^3^VAS – Visual Analogue Scale (range 0–10 cm)

^4^HAQDI – Health Assessment Questionnaire Disability Index (range 0−3)

^5^BRAF MDQ—Bristol Rheumatoid Arthritis Fatigue Multidimensional Questionnaire (range 0–70)

^6^TPB – Theory of Planned Behaviour Questionnaire

^7^RA QoL – Rheumatoid Arthritis Quality of Life Scale (range 0–30, higher scores indicate worsening QoL)

^8^PSQI – Pittsburgh Sleep Quality Index (range 0–21, higher scores indicate worse sleep quality)

#### Acceptability of the intervention

Interviews were undertaken with 12 patients (4 intervention and 8 control), with 6 HCPs (3 sessional physiotherapists, 2 rheumatologists and 1 rheumatology nurse). Following the transcription of all interviews, each transcript was read and re-read by one member of the research team (AF). Using content analysis segments of the transcripts were coded and grouped in the subsequent stages of analysis where the initial sets of codes were reduced down into summary form to form subcategories.

Several categories were identified in relation to the factors which prompted acceptability of this intervention for the participants (Table 3) and the HCPs (Table 4). The categories identified from the participant interviews were (i) positive effect of outcome measures to participation (ii) acceptability of information (iii) physiotherapist as a suitable guide (iv) recruitment process to be face-to-face and the (v) positive effect of social interactions on physical activity habits. The sessional physiotherapist and HCPs interviews discussed a number of additional categories: i) the intervention setting ii) resources available to support the intervention iii) enhanced communication and enhancements to recruitment. Overall, across both groups the intervention and outcome measures were acceptable, with suggestions made to enhance recruitment and intervention delivery.

**Table 3:** Themes and supporting quotes from patient interviews

Themes	Sub-themes	Supporting quote
Outcome measures	PA devices	The wearable health monitor was accepted by the patients as it did not intrude on their daily lives. It was commonly reported by patients that they were unaware of the monitor’s presence once applied *P003- “I didn’t know most of the time that I was even wearing it…”* *P005- ‘’ I think from the first time it didn’t feel like I had it on me. Yes, it was very comfortable to use.’’P016- ‘’Yes, no problem. Just like I said I didn’t even think about it. I put it on and I didn’t think about it all day long.’’*
Acceptability of study information		This leaflet was reported by staff and patients as being an efficient tool to explain aspects of the participation and details of physical activity, appreciation towards a simple and concise structure *P035- ‘’ That was pretty informative you know I mean I k new what I was letting myself in for at that stage.’’* *‘’But when I read that I said you know this can’t be bad.’’* *P004- ‘’Yes it did tell me a small bit about the study. Obviously, there’s a lot more that I don’t know about this arthritis you know like I say the physical part was knowledgeable when it comes to cramps and that, the exercises, breathing, coming out having to do walks to do the walks regular which I didn’t know.’’* *P028- ‘’It kind of explained everything yes and it made a lot of sense.’’* *‘’ So it’s important to make these things accessible to patients, not too detailed, not too long, but also enough information to make it functional. I thought it worked very well.’’* Amongst this cohort of patients, it was commented that possession of the information leaflet prompted some to revise physical activity information and continue the exercises *P006- ‘’ It was helpful because it was handy to refer to something.’’* *‘’It reminds you oh, I must try that one kind of thing, do you know what I mean?’’* *P033- ‘’ But when I thought about it like I said sure I have a few leaflets there from the physiotherapist and I’ll do that.’’*
Physiotherapist as a suitable guide		It was identified from patient interviews that they valued the physiotherapist as a trustworthy source of information to allow them to be comfortable taking part in physical activity *P003- ‘’I think it helps the physio would help people be more active because they know exactly what they can and can’t do.’’* *P004- ‘’ I had someone there to tell me what to do, the physio was there that gave me more information on how I should go about doing these workouts without hurting myself or causing any problems. So yes it was good.’’* *‘’So you know what to do, I had all the information from the physio and what to do from that aspect, so yes it’s very informing yes, it’s good.’’* It was important that the physiotherapist met the patients need of having a good understanding of their specific disease management *P006—‘’You wouldn’t mind so long as you thought they knew what they were doing, and they understood the problem.’* It was also noted that way in which the physiotherapist implemented the physical activity intervention, utilising behaviour change techniques, impacted the patients in a positive way to allow them to view the physiotherapist as a suitable guide *P006- ‘’ She kind of explained it before she’d even ask you to try doing it and then it was try rather than do.’’* *‘’She seemed to understand and would say try it rather than do it which was good.’’* *P028—‘’So that was, I think, probably the most beneficial part of the whole thing kind of having an in depth discussion about what you would find specifically difficult and have things specifically tailored to you.’’*
In-person recruitment		To be approached by staff personally in the clinic positively resonated with the patients. This method of recruitment was effective in creating a motivated attitude and they felt they were given clear information to prompt them to participate *P033- ‘’ She explained to me nice and clearly what it was about. She gave me a leaflet, I think I went into a room and had time to read it and said yes, you can put me down for that.’’* *P054- ‘’ No, no I found you gave great information, yes.’’* *P053- ‘’But I must say that it was explained very well but I had no hesitation whatsoever taking part with the study.’’*
Value of social interaction on PA habits		This theme suggests ways for enhancing the PIPPRA study in highlighting how a social atmosphere affected their ability to engage with physical activity *P006- ‘’When you are not going out it’s hard to get your head in terms of I need to go walking- But it’s amazing when you are not going to town you think you won’t be sore because I won’t be walking around and you take things easier.’’* It was also expressed by patient and physiotherapist for future considerations to include group-based classes *P025- ‘’ if there was a lot of people around west Limerick area involved that would go to a pool and do an easy exercise class’’* *Physio 1 – ‘’ But like some people would have I suppose done better in a group situation if it was exercise based’’* *‘’-like having four sessions would be great and then allowing people to go into a class. You know, because that then would enable people, the patients to do strengthening and exercises in a class setting and that would only be beneficial for them with peer support.’'*
Further design considerations	Setting	Patients expressed their difficulty managing their physical activity habits during the COVID pandemic which disrupted the timeline of this intervention. The period of isolation showed to cause emotional distress to some of the patients *P035- ‘’ I found during COVID there was a lot of times I really got a bit I won't say depressed it's not the word I would use but you could see look is there ever going to be an end to this.’’* *P054- ‘’ I find covid really took it out of people you know because you couldn’t see your families, you couldn’t go anywhere’'* Additionally, patients found this site to challenge their time management due to lack of consistent parking availability *P024- ‘’-but it was more hassle trying to park the car, trying to allow time.’'* Multiple patients expressed disinterest or personal difficulties when accessing content virtually and using technology
	Assessment length	*P006- ‘’No, I'm not into computers at all.’’* *P016-’Sometimes I have a problem understanding people on the phone and that’s why I prefer face to face.’’* *‘’And I think it’s better if you see the other person that you are talking to.’’* *P028- ‘’ I don’t think to be honest doing it online would be really good at all’’* From one patient's perspective they found that the number of questions asked at assessments affected them negatively, as they felt it was not helpful at times when they felt they were at their best *P004- ‘’ Annoyed I’ll be honest with you there were a lot of questions / Especially when I am doing well at the moment some of the questions asked didn’t really matter at the time.’’*

**Table 4:** Themes and supporting quotes from health care staff professionals

Theme	Sub-theme	Supporting quote
Setting		Regarding the setting for the intervention the sessional physiotherapists views were to deliver it outside the hospital would be preferable due to their own inexperience in that setting and preference for a private practice facility *Physio 1- ‘’ I suppose my background is in private practice, so it would have been easy you know, that’s easy for me to say that it would have been easier in private practice. Like because the parking is straight outside and you have access to goniometers and other objective measures, handheld dynamometers, and if people had weights or wanted to use weights then they could, but you could keep it simple for the people who didn’t want to use any of that stuff, you know.’’ ‘’ And I’m sure if I had worked in a hospital, that might be easy as well, you know, because it’s not going to be hugely different in private practice other than you probably have less time and less parking.’’*
Resources available		It was highlighted how access to a wider range of equipment could have the potential to track progress more effectively with outcome measures, as well as give patients a better understanding of how to correctly carry out the specific exercise *Physio 1- ‘’It might have been good to have maybe grip things, handheld dynamometers, you know those types of things just to see….but it might have been nice to have an objective measure.’’* *‘’RPE scale might have been beneficial actually, or a treadmill or something for them to go – because we couldn’t go out for a walk and go you need to work at this pace, you know so even a treadmill or a bike or something to just show them this is where they need to be working.’’* As well as this, physiotherapists expressed how having access to different educational tools including visual and audio resources would be beneficial for the implementation of the patient’s exercise at home *Physio 1- ‘’…having a database of exercises to be able to print out for them or send by email.’’* *Physio 2- ‘’If there was some kind of generic, maybe that’s in the initial leaflet that a person gets, I am not sure. Again, for each session if there is a little bit of supplementary leaflet, or again if there's a video type thing that’s good, or anything like that, for some people they might prefer that. They could supplement some of the kind of key messages there on each session so that the person has that to refer to maybe.’’* *‘’That there is a sheet where we write down the goals together if I am face-to-face with the person and they take that away and bring it back with them the next time we go through it. It’s almost like a contract, if you like.’’* *‘'…having pictures and having videos to help people learn and so forth, there is a huge value that they’ll have a better technique and better outcome if they have the support of that material as well.’’*
Areas for further consideration	Communication between sessional physiotherapists	The physiotherapists suggested ways to have communication between the sessional physiotherapists when planning future interventions to enable peer support as well as aid in creating a good understanding of how they should aim to structure each session *Physio 1- ‘’ I think it would be good for them to be able to talk to one another in terms of what worked for certain people and what didn’t work for certain people.’’*
	Pre-study preparation	*Physio 2- ‘’I did actually shadow or observe a session with one of the other physios as well as beforehand, so again just to kind of give me a sense and a feel of what was involved.’'*
	Access to patients baseline information	*‘’Will people get together and kind of a little bit feedback amongst themselves or maybe with one of the people running the trial or something like that, that could be useful just to ensure that people are feeling like they are on the right track with delivering it maybe.’’*
	Time between sessions	It was suggested that improvements could be made to the resources provided to the physiotherapists prior to the commencement of the intervention *Physio 2- ‘’video-type resources sometimes it’s just easier’’* *‘’…even if it’s Youtube, something like that, video examples of that kind of communication approach demonstrated just to make a bit more real life.’’* To receive the patient’s baseline information before the physiotherapist sees them for the first time was thought to allow for more time to be spent on aspects of behaviour change, improving time management.7
	Recruitment of patients	*Physio 2- ‘’ It allows you a little bit more time on the first session and even in subsequent sessions to focus more on the motivational interviewing approach and that behaviour change approach, because I felt a bit squeezed one time to some degree on those aspects…’’ ‘’ So having that information in advance can kind of even help to structure a little bit of the subjective part as well, you know.’’* Consideration should be given to whether it would be more effective to monitor skill retention and habit formation if there was more time placed in between each physiotherapy session *Physio 2- ‘’ I think sometimes over eight weeks do we really get enough of a change that will sustain behaviours and so forth and I wonder about whether, know, something, maybe it’s five or six sessions but some certainly from sessions 3,4,5 or whatever it might be, there might be a bigger gap between them. So you are kind of then able to judge, okay, they have done two weeks. They have done two weeks. Now they do three weeks, Now they do four weeks. Have they been able to sustain those behaviours over a longer period of time? And you’d have, I suppose, personally I feel more confident that they will actually sustain them beyond the intervention, if that makes sense.”*
		The recruitment method used was acceptable to healthcare staff as they were familiar with these patients and appreciated this method over their electronic resources *HCP 1—‘’ I didn’t feel that it was a difficult task to do at the clinic and most of the patients, I know that they agreed.’’* *‘’ So I think the best way in my opinion is through clinics because we don’t have a very good electronic database just to pick up the patients, if we want, off site.’’* Prescreening was mentioned for future considerations as it could improve efficiency of the recruitment process *HCP 2*— *‘’So whatever the cohort of patients that you were looking for, if we pre-screened to make sure that we have whoever on the list and put it your questionnaire then on your, you know, with your patient files so that the medical doctor, or nurse, or whoever, picks it up knows that that patient is suitable for the trial and follow on with the questions then. I think the prepping of the clinic would make an awful difference.’'* It was suggested that the placement of a study representative in the clinic could assist staff in the future in relation to patient’s specific questions regarding details of the intervention *HCP 2*— *‘’ Well it is helpful because if there is anything, you know, some patients might want to discuss something further and maybe something, having the clinical person there is helpful.’’*

#### Progression to full scale trial

Three criteria were stated at the outset to determine if a future definitive trial would be feasible. The stated recruitment rate of 4 participants per month was exceeded (6.4 per month). The second criterion was a minimum average attendance by recruited participants of 100% of the intervention sessions. This criterion was not met for all 4 sessions (intervention group participants completed 100% of session 1 & 2, 88% session 3 and 81% session 4). The final criterion was a minimum retention of 80% of recruited participants providing valid 12-week outcome data. This was not met as the percentage of participants who completed assessments at T1 was 53% and 69% at T2.

#### Power and sample size calculations for future definitive intervention

The standard deviation of the step count was calculated at approximately 2500 steps, hence, to detect a difference of at least 1000 steps in a double-armed trial, at a 5% level of significance and with an 80% power, a sample of size of 200 (balanced design, 100 in each group) will be required in a future full-scale clinical trial.

## Discussion

The study objectives were to assess the feasibility and acceptability of a BC intervention in improving PA in people with RA who were physically inactive. The study achieved its objectives on feasibility and determined a recruitment rate of 6.4 participants per month. We exceeded the target sample size, but had to exclude a large number of participants from the data analysis due to the impact of COVID-19 restrictions on access to clinics for recruitment and treatment rooms for intervention delivery and assessments. A commonly reported issue with the conduct of RCTs is that recruitment is often slower or more difficult than expected [37, 38]. However, the PIPPRA study achieved a higher than expected recruitment rate from a single rheumatology unit indicating the feasibility of the recruitment in a larger study. The restrictions also impacted the achievement of the minimum outcome assessment target.

We were also able to calculate the sample size for a definitive intervention using data from other studies as well as PIPPRA to address concerns on the high variability of the PIPPRA data. The secondary measures were practical to incorporate into the study design and even with the change to virtual delivery and assessment only one outcome measure (DAS28) had to be removed. As this study was a feasibility trial the secondary outcome measures were completed for the purpose of estimation of standard deviations only and to inform sample size calculations. No conclusion on the effectiveness of the interventions can be drawn at this feasibility stage. Although only one of the 3 pre-set criteria for progression to full trial were met, the two that were not met (attendance at 100% of intervention sessions and 80% of recruited participants providing valid 12-week outcome data) were impacted by COVID-19 related issues. Given the pandemic context the actual results for these 2 criteria still achieved good results despite the circumstances (88% session 3 and 81% session 4 completion and completion of assessments at T1 was 53% and 69% at T2).

The PIPPRA study was considered acceptable to the participants, the sessional physiotherapists and the rheumatology health care professionals involved in recruitment and ongoing usual care for the participants. Enhancements to the design that were suggested included allowing more time for change in behaviour to be seen by extending the time between sessions 3 and 4. Establishing a way for the sessional physiotherapists to communicate with each other before and during the study was also proposed. A desire for more baseline information as would be standard in clinical care as well as a greater breadth of resources to include visual as well as written materials to support intervention delivery were also suggested as enhancements. From the patient perspective the outcome measures were considered acceptable with some participants advising on the need to consider the length of time the assessments took as it was too onerous. Participants valued the physiotherapists guidance reinforcing the need for physiotherapist interventions to improve physical activity. There was not universal agreement that virtual intervention only was the preferred choice suggesting the option of in person and/or virtual delivery should be considered in future interventions. This resonates with other research [39] which evaluated in person and videoconferencing delivery of an education programme for people with inflammatory arthritis and found no difference in outcomes between the two modes. Similarly, a study [40] on the perspectives of people with Psoriatic Arthritis on virtual consultation during COVID-19 found a wide range of perspectives on its benefits. The value of the social side of exercise in future interventions should also be considered to add to the experience and enjoyment of the intervention.

Therefore, the data from this study indicates that the PIPPRA intervention was both feasible, acceptable to and safe for people with RA who were physically inactive.

The PIPPRA study pilot trial methods provide valuable learnings for others designing trials of interventions in lifestyle/behaviour change areas for a range of cohorts, including non-clinical populations. The inclusion of a screening tool (GSLPAQ) to target people with RA who were physically inactive is a valuable addition to the literature on improving PA in people with inflammatory arthritis. A criticism of previous studies aiming to improve PA levels has been that participants included were already engaged in some level of PA and targeting those with low or now PA profile is important. Hence the PIPPRA study was designed to only include those with low levels of PA. The GSLPAQ was a useful tool in screening—a future study should use an additional tool alongside the GSLPAQ to explore the sensitivity of the GSLPAQ.

### Study limitations

Participants in this study were independently mobile and able to be active and therefore are not representative of the total RA population particularly those with greater mobility limitations and with a variety of activity levels. Hence, a selection bias in the sampling should be noted.

The sample size of 40 patients was not reached due to the interruption of COVID-19 on the study. While the study’s feasibility objectives were still met the results on the secondary objectives need to be interpreted with caution.

As the study examined an intervention delivered by a physiotherapist compared to a control intervention of a PA leaflet, it was not possible to blind participants to the intervention. This is an accepted design limitations of this type of intervention.

The study was designed to consider pragmatic aspects of delivery of this type of care in healthcare settings including delivery by clinicians who are not experts in behaviour change. This presents a possible source of bias in differences in how the sessional physiotherapists who delivered the intervention. As this is a feasibility study the effectiveness of the intervention was not the primary focus and hence this possible bias does not impact the study results.

COVID-19 disrupted the study in many ways [20] and as described previously—complete data sets were not available for all participants enrolled pre COVID-19. Overall, despite the impact of COVID-19, there are no outstanding uncertainties relating to the feasibility of the study.

The number of interviews with participants involved less participants from the intervention than the control arm. Despite attempts to increase the number of intervention participants it was not possible due to the timing of the end of the study.

## Conclusion

The PIPPRA study to improve promote physical activity in people with RA who have low PA levels was feasible, acceptable and safe. Despite the impact of COVID-19 on the recruitment and retention of patients, the study provides preliminary evidence that this physiotherapist led BC intervention is feasible and a full definitive intervention should be undertaken. Health care professionals involved in the study delivery and the patient participants described a number of positive aspects to the study with some suggestions to enhance the design. These findings hence inform the design of a future efficacy-focused clinical trial.

### Supplementary Information

Below is the link to the electronic supplementary material.

Supplementary file1 (PDF 2053 KB)

Supplementary file2 (PDF 329 KB)

Supplementary file3 (PDF 131 KB)

Supplementary file4 (PDF 335 KB)

Supplementary file5 (PDF 144 KB)

## Data Availability

The data underlying this article will be shared on reasonable request to the corresponding author.
