# Supplementary material for: Promoting physical activity in rheumatoid arthritis through a physiotherapist led behaviour change-based intervention (PIPPRA): a feasibility randomised trial
Source: Rheumatol Int. 2024 Mar 4;44(5):779–93. doi: 10.1007/s00296-024-05544-1 (PMC10980645; doi:10.1007/s00296-024-05544-1)
Supplement: Supplementary file 1 — Supplementary file1 (PDF 2053 KB) [file 296_2024_5544_MOESM1_ESM.pdf]

PIPPRA

A **P**hysiotherapist led **I**ntervention to **P**romote **P**hysical activity in **R**heumatoid **A**rthritis

Contents

Introduction to PIPPRA 3

How to use the manual 4

Rheumatoid Arthritis 6

Physical Activity Recommendations for People who have RA 7

Overview of PIPPRA Intervention 10

Physical activity information 10

Session 1 11

Session 2 11

Session 3 11

Session 4 12

Intervention detail log sheet 13

Delivering the intervention 14

Supporting physical activity – resources 15

Appendices 16

Appendix 1: Behaviour Change Taxonomy (v1) Michie et al (2013) 16

Appendix 2: Behaviour Change Taxonomy (v1) Michie et al (2013) – short version 45

Introduction to PIPPRA

The PIPPRA (**P**hysiotherapist led **I**ntervention to **P**romote **P**hysical activity in **R**heumatoid **A**rthritis – a pilot study) is a Health Research Board funded project that will examine physical activity in people with rheumatoid arthritis (RA).

The aim of this pilot randomised controlled trial (RCT) is to examine the feasibility of a physiotherapist-led, behaviour change theory-informed, physical activity intervention to promote physical activity in people who have RA who have low levels of physical activity. The study will work out the practicalities of doing the RCT before proceeding to do it as a larger study in different rheumatology centres in Ireland. This first step is important to see how the intervention works, how easy it is to recruit people with RA to take part and to see what participants think of the intervention and the outcome measures used.

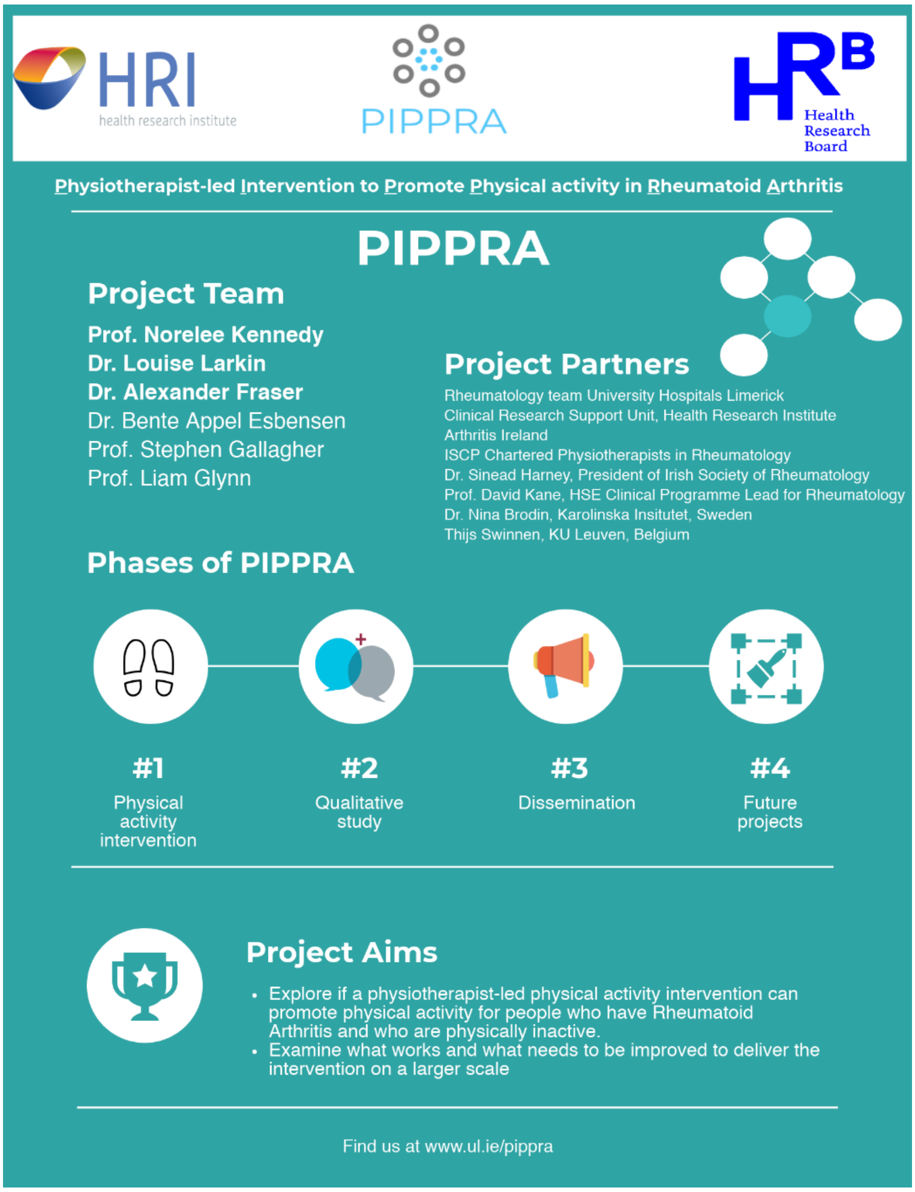

The study will be completed in three work packages (WP).

- WP1 is the pilot RCT study, which will be delivered at University Hospitals Limerick.
- WP2 is the qualitative acceptability study, where participants and all involved with the pilot RCT delivery will be interviewed to find out what worked and what could be improved. This will help to guide the delivery of the RCT on a larger scale in the future.
- WP3 is the dissemination and future planning phase. The findings of WP1 and WP2 will be published in research journals and presented to conferences and wider audiences.

How to use the manual

This manual provides information on how to deliver the PIPPRA intervention.

It provides a brief overview of rheumatoid arthritis (RA), physical activity and recommendations for being physically active in RA, and details on the four sessions of the PIPRA intervention.

The intervention is theoretically informed and was designed, using the Behaviour Change Wheel and the Theory of Planned Behaviour.

The Behaviour Change Wheel (BCW) was developed from 19 frameworks of behaviour change identified in a systematic literature review.

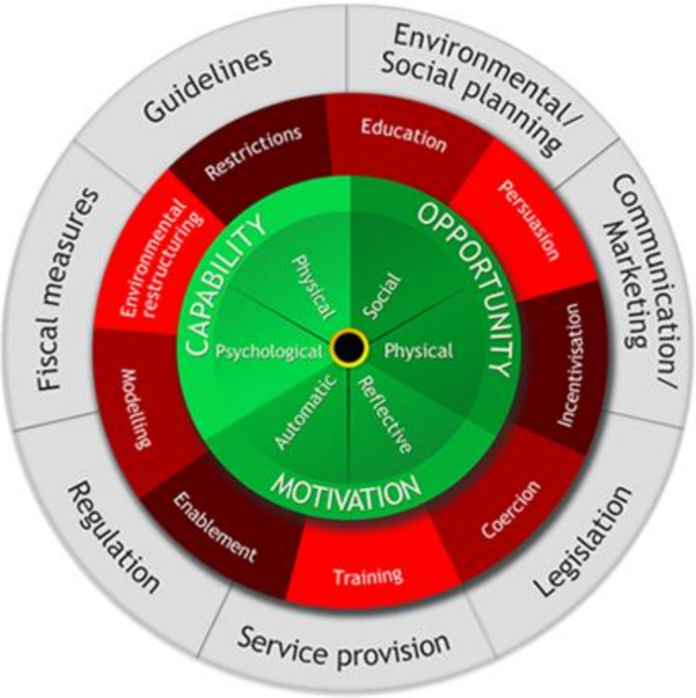

It consists of three layers. The hub identifies the sources of the behaviour that could prove fruitful targets for intervention. It uses the COM-B ('capability', 'opportunity', 'motivation' and 'behaviour') model. This model recognises that behaviour is part of an interacting system involving all these components. Interventions need to change one or more of them in such a way as to put the system into a new configuration and minimise the risk of it reverting. Surrounding the hub is a layer of nine intervention functions to choose from based on the particular COM-B analysis one has undertaken. The outer layer, the rim of the wheel, identifies seven policy categories that can support the delivery of these intervention functions.

Further information is available here: http://www.behaviourchangewheel.com/about-wheel

The Theory of Planned Behaviour (Azjen 1991) is a beliefs/attitude-based theory, which focuses on an individual's intention to perform a given behaviour. Intentions are assumed to capture the motivational factors that influence a behaviour; they are indications of how hard people are willing to try, of how much of an effort they are planning to exert, in order to perform the behaviour. As a general rule, the stronger the intention to engage in a behaviour, the more likely should be its performance. The TPB postulates three conceptually independent determinants of intention:

1. Attitude: attitude toward the behaviour and refers to the degree to which a person has a favourable or unfavourable evaluation or appraisal of the behaviour in question
2. Subjective Norm: Subjective norm is a social factor it refers to the perceived social pressure to perform or not to perform the behaviour
3. Degree of Perceived Behavioural Control: refers to the perceived ease or difficulty of performing the behaviour and it is assumed to reflect past experience as well as anticipated impediments and obstacles

Overview of Theory of Planned Behaviour

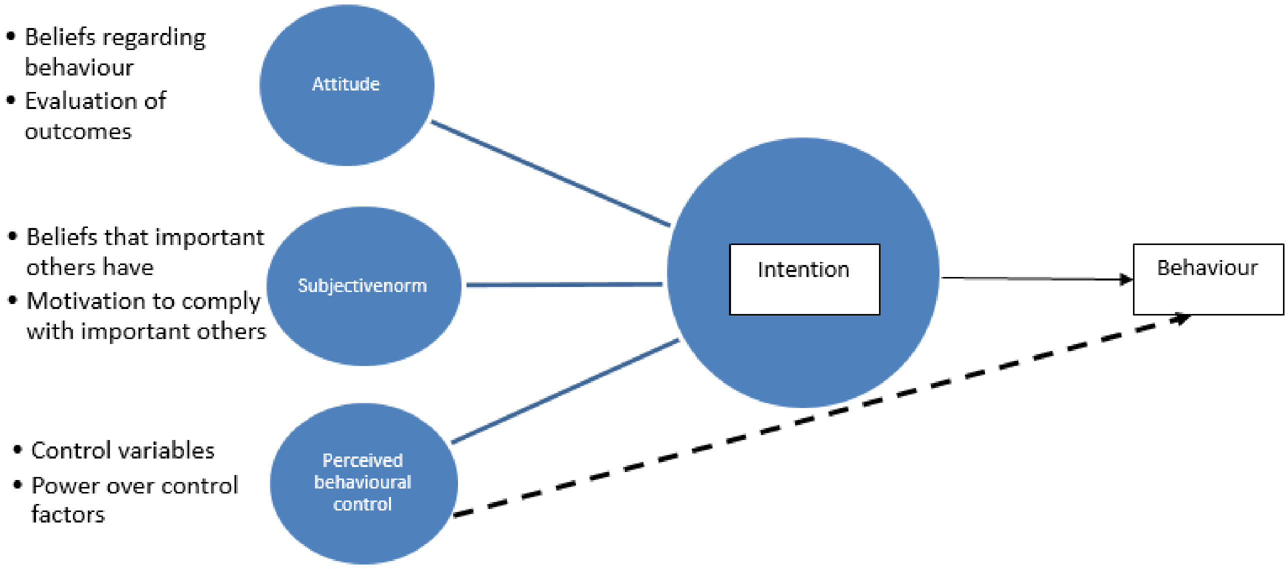

Further information is available here
https://www.sciencedirect.com/science/article/pii/074959789190020T

Rheumatoid Arthritis

Rheumatoid arthritis (RA) is a chronic inflammatory, autoimmune disease characterised by joint swelling, joint tenderness, and destruction of synovial joints. This can lead to severe disability and premature mortality. Between 1 and 3 per hundred develop rheumatoid arthritis and approximately 40,000 people in Ireland have RA. The incidence of RA is varied in that it can occur at any age but peak age of onset is between 30 and 50 years. Women are affected 3 times more than men. RA is diagnosed based on the American College of Rheumatology (ACR)/ European League Against Rheumatism (EULAR) criteria (see Figure 1).

Figure 1: Diagnostic criteria for RA

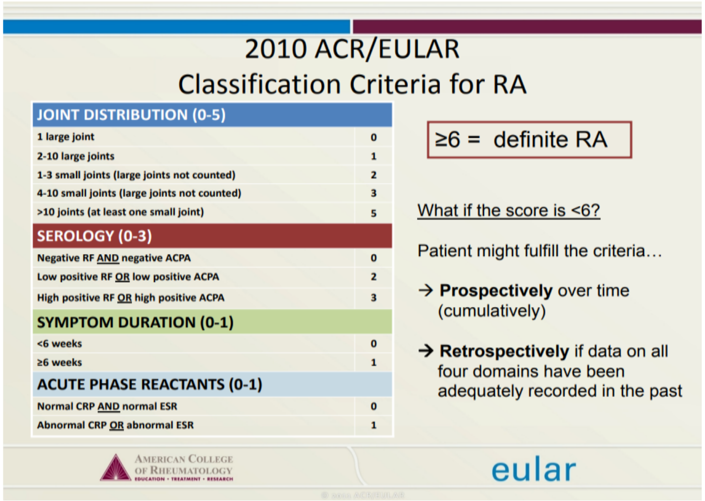

People who have RA experience many symptoms including:

- Pain
- Tenderness
- Swelling
- Heat
- Erythema
- Joint deformity
- Muscle Wasting
- Decreased range of motion
- Stiffness/Early Morning Stiffness
- Loss of function
- Fatigue
- Poor sleep
- Mental health impact, i.e. anxiety, depression

In addition to the above symptoms people who have RA at a higher risk of certain comorbidities, due to the systemic inflammation in this condition. Pro-inflammatory cytokines are involved in many disease processes, e.g. joint destruction, altered body composition (rheumatoid cachexia) and changes in vascular function. This systemic inflammation results in an increased incidence of cardiovascular disease in people who have RA and can also impact pulmonary function, e.g. pleural effusions, interstitial

lung disease and fibrosing alveolitis which causes chronic cough & dyspnoea (Metsios et al 2010; Bongartz et al 2010). The skin and eyes may also be affected.

Management

Pharmacological management is the first line of treatment, with lifestyle management (smoking cessation, physical activity, healthy eating) playing an important role in maintaining and improving health outcomes and quality of life (ACR 2015, EULAR 2016).

Common medications (oral/joint injection/biologic infusions)

- Analgaesics, e.g. Paracetemol
- Nonsteroidal Anti-Inflammatory Drugs (**NSAIDs**), e.g. Diclofenac (Votarol), Naproxen (Naprosyn) and Piroxicam (Feldene)
- Disease-Modifying Antirheumatic Drugs (**DMARDs**), e.g. Methotrexate
- **Corticosteroids** orally, via infusion or injection, e.g. Methylprednisolone, Prednisolone
- **Biologics** come as injections and can also be given intravenously. e.g. Adalimumab, Etanercept, Infliximab, Enbrel & Humira

Physical Activity Recommendations for People who have RA

Physical Activity Recommendations

Type	Recommendation
**Aerobic**	**At least**<br>**150 minutes** of moderate intensity physical activity on per week<br>OR<br>**75 minutes** of vigorous-intensity aerobic physical activity a week
**Strength**	Muscle **strengthening** activities of all major muscle groups on<br>**2 or more days** per week
**Flexibility**	Flexibility exercises **2-3 days** per week
**Neuromotor**	Balance, agility, coordination, and gait at least 2 to 3 days per week

Exercising during a flare-up

Flare-ups (an acute exacerbation of symptoms) occur in inflammatory conditions. During a flare-up physical activity should be modified to the preference of the person who has RA. Physical activity should not be stopped completely during a flare-up, rather should be modified to accommodate the increase in symptoms, with a gradual return to prior physical activity levels once the flare-up has resolved.

EULAR 2018 Recommendations

Table 2 Recommendations for PA and exercise in people with inflammatory arthritis and OA

Overarching principles

1. PA is part of a general concept to optimise health related quality of life.
2. PA has health benefits for people with RA/SpA/HOA/KOA.
3. General PA recommendations, including the four domains (cardiorespiratory fitness, muscle strength, flexibility and neuromotor performance) are applicable (feasible and safe) to people with RA/OA/SpA.
4. The planning of PA requires a shared decision between healthcare providers and people with RA/SpA/HOA/KOA, which takes people's preferences, capabilities and resources into account.

Recommendations	Category of evidence	Strength of recommendation	Level of Agreement mean (SD) Median (Range)
1. Promoting PA consistent with general PA recommendations should be an integral part of standard care throughout the course of disease in people with RA/SpA/HOA/KOA.	1B	A	9.81 (0.39)<br>10 (9–10)
2. All healthcare providers involved in the management of people with RA/SpA/HOA/KOA should take responsibility for promoting PA and should cooperate, including making necessary referrals, to ensure that people with RA/SpA/HOA/KOA receive appropriate PA-interventions.	4	D	9.14 (0.98)<br>9 (7–10)
3. PA interventions should be delivered by healthcare providers competent in their delivery to people with RA/SpA/HOA/KOA.	4	D	8.86 (1.48)<br>10 (5–10)
4. Healthcare providers should evaluate the type, intensity, frequency and duration of the people's actual PA by means of standardised methods to identify which of the four domains of general PA recommendations can be targeted for improvement.	3	C	9.05 (1.04)<br>9 (6–10)
5. General and disease-specific contraindications for PA should be identified and taken into account in the promotion of PA.	4	D	9.10 (1.41)<br>10 (5–10)
6. PA interventions should have clear personalised aims, which should be evaluated over time, preferably by use of a combination of subjective and objective measures (including self-monitoring when appropriate).	4	D	9.05 (1.25)<br>9 (5–10)
7. General and disease-specific barriers and facilitators related to performing PA, including knowledge, social support, symptom control and self-regulation should be identified and addressed.	3	C	9.19 (1.13)<br>10 (6–10)
8. Where individual adaptations to general PA recommendations are needed, these should be based on a comprehensive assessment of physical, social and psychological factors including fatigue, pain, depression and disease activity.	4	D	9.24 (0.86)<br>9 (7–10)
9. Healthcare providers should plan and deliver PA interventions that include the behavioural change techniques self-monitoring, goal setting, action planning, feedback and problem solving.	1A	A	9.48 (0.79)<br>10 (7–10)
10. Healthcare providers should consider different modes of delivery of PA (eg, supervised/not-supervised, individual/group, face-to-face/online, booster strategies) in line with people's preferences.	4	D	9.00 (1.30)<br>9 (5–10)

HOA, hip osteoarthritis; KOA, knee osteoarthritis; OA, osteoarthritis; PA, physical activity; RA, rheumatoid arthritis; SpA, spondyloarthritis.

Key Reference: Rausch Osthoff A, Niedermann K, Braun J, *et al* 2018 EULAR recommendations for physical activity in people with inflammatory arthritis and osteoarthritis *Annals of the Rheumatic Diseases* 2018;**77:**1251-1260.

Benefits of Physical Activity in RA

Physical activity has many physical, psychological and social benefits for people who have RA. Physical activity reduces the symptoms associated with RA, including pain and fatigue, and disease-related characteristics, including joint damage. Furthermore physical activity has been highlighted as an essential component in reducing the risk of cardiovascular disease by the European League Against Rheumatism (EULAR) recommendations for cardiovascular risk management in people with rheumatoid arthritis (Peters et al. 2010). Being physically active also benefits people who have RA from a psychological and social perspective. People who have RA have reported that

Benefits of Physical Activity in RA continued

being physically active is empowering and it demonstrates that they have taken responsibility for their own health. Others have reported that being physically active improved their mental health. The social interaction linked with being physically active is reported to be enjoyable for people who have RA. All of this indicates that participating in regular physical activity has been shown to benefit the overall health and wellbeing of people who have RA.

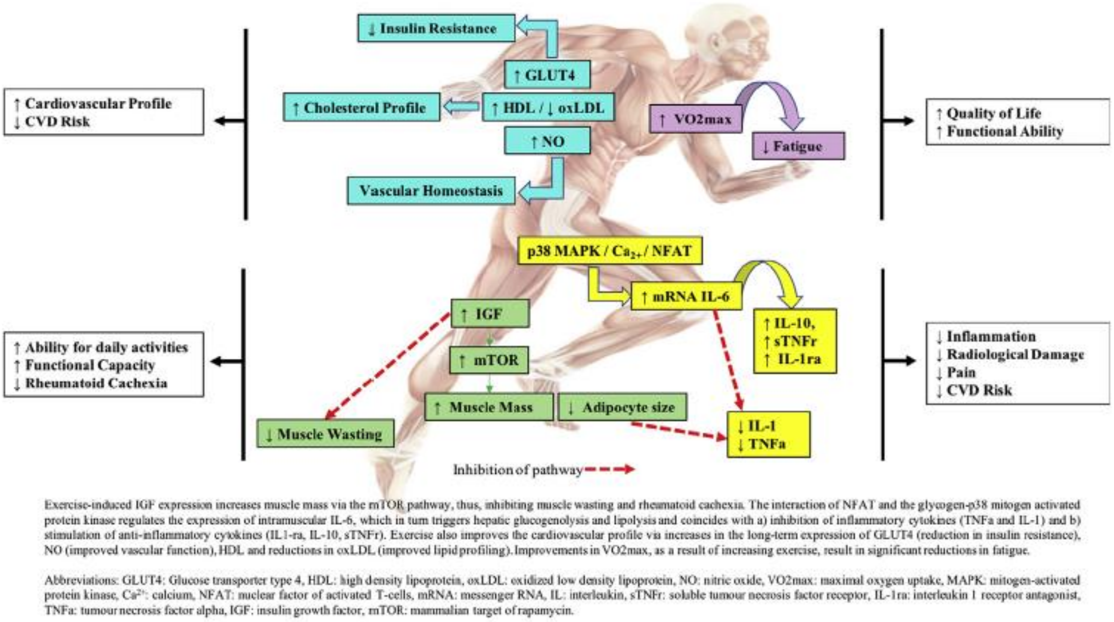

Exercise-induced IGF expression increases muscle mass via the mTOR pathway, thus, inhibiting muscle wasting and rheumatoid cachexia. The interaction of NFAT and the glycogen-p38 mitogen activated protein kinase regulates the expression of intramuscular IL-6, which in turn triggers hepatic glucogenolysis and lipolysis and coincides with a) inhibition of inflammatory cytokines (TNFa and IL-1) and b) stimulation of anti-inflammatory cytokines (IL1-ra, IL-10, sTNFr). Exercise also improves the cardiovascular profile via increases in the long-term expression of GLUT4 (reduction in insulin resistance), NO (improved vascular function), HDL and reductions in oxLDL (improved lipid profiling). Improvements in VO2max, as a result of increasing exercise, result in significant reductions in fatigue.

Abbreviations: GLUT4: Glucose transporter type 4, HDL: high density lipoprotein, oxLDL: oxidized low density lipoprotein, NO: nitric oxide, VO2max: maximal oxygen uptake, MAPK: mitogen-activated protein kinase, Ca^{2+}: calcium, NFAT: nuclear factor of activated T-cells, mRNA: messenger RNA, IL: interleukin, sTNFr: soluble tumour necrosis factor receptor, IL-1ra: interleukin 1 receptor antagonist, TNFa: tumour necrosis factor alpha, IGF: insulin growth factor, mTOR: mammalian target of rapamycin.

(Metsios and Kitas 2018)

Safety of Physical Activity in RA

Physical activity and exercise are safe!

- Physical activity is an integral part of standard care throughout the course of disease in people with RA, given its effectiveness, safety and feasibility (Rausch Osthoff et al 2018)
- High-intensity, low-impact aerobic and resistance exercise safe without change in markers of bone health (Law et al 2015)
- Aerobic and strengthening exercises have no harmful side-effects (Cochrane Review - Hurkmans et al 2009)
- Aerobic and strengthening programmes for people who have RA are effective and safe (Kennedy et al 2006)

Overview of PIPPRA Intervention

The PIPPRA intervention consists of 4 1:1 sessions with a physiotherapist over seven weeks. Participants will work with the physiotherapist to become more physically active. Participants will continue with their routine care for their condition throughout the study period.

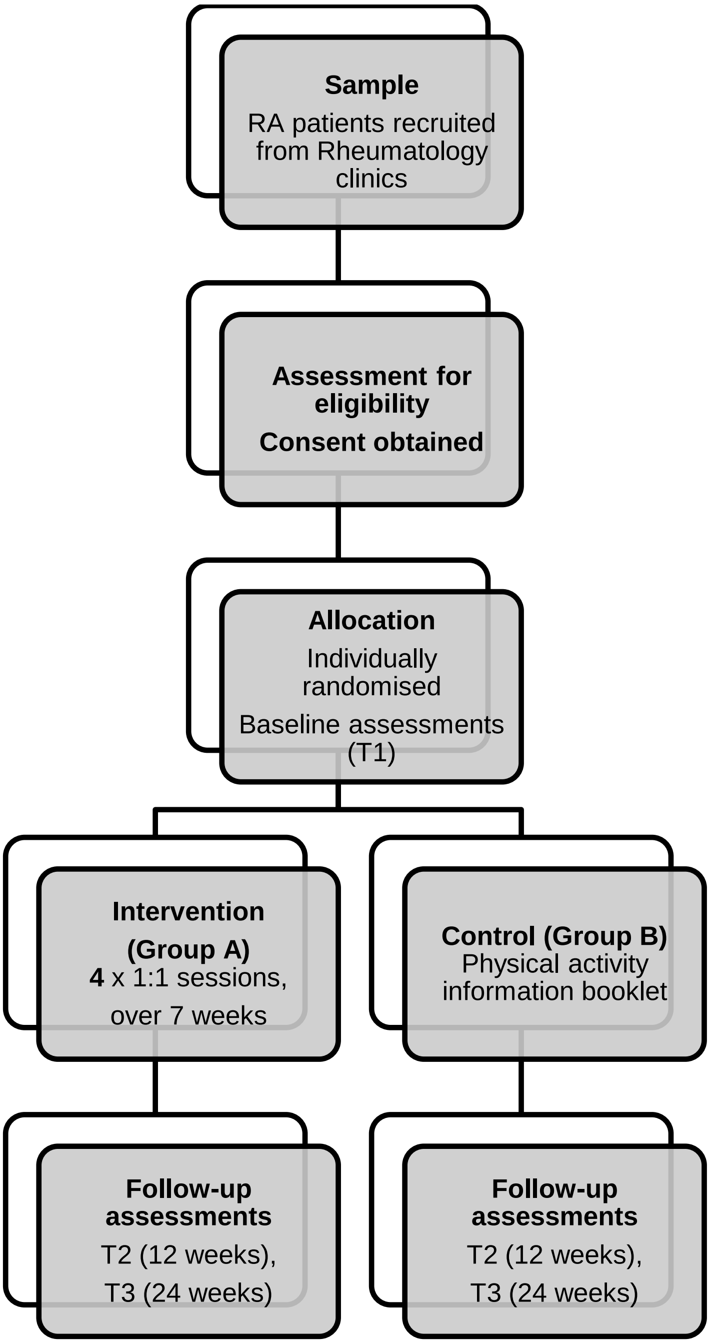

Physical activity information

Participants will be provided with a physical activity information leaflet, outlining the recommendations and benefits of physical activity. The leaflet will be given to participants at their baseline assessment by the assessing physiotherapist. Participants will be given a diary to note their activities over the intervention period.

Session 1

Session one (week 1) will consist of specific behaviour change techniques, as described by the Behaviour Change Taxonomy (Michie et al 2013).
This session should incorporate 'education' and enablement' techniques.
A physical activity behaviour plan should be developed in this session.
Participants should be advised to continue this physical activity plan outside of intervention sessions.

**Session number & time point**	**Suggested Content, including behaviour change techniques**
Session 1<br>Week 1	***Education techniques***<br>4.1 instruction on how to perform a behaviour<br>5.1 information about health consequences<br>5.2 salience of consequences<br>***Enablement techniques***<br>1.1 goal setting (behaviour)<br>1.2 problem solving<br>1.4 action planning
Session outcome	Physical activity plan developed<br>Physical activity plan implemented independently outside of intervention sessions

Session 2

Session two (week 3) will focus on 'enablement' techniques. Participant physical activity plan will be reviewed and adjusted as necessary to ensure a graded, progressive approach is taken across the intervention.

**Session number & time point**	**Suggested Content, including behaviour change techniques**
Session 2<br>Week 3	***Enablement techniques***<br>1.5 reviewing behaviour goals<br>***Modeling techniques***<br>2.2 feedback on behaviour<br>2.3 self-monitoring of behaviour<br>6.1 demonstration of behaviour<br>6.2 social comparison<br>8.1 behavioural practice/rehearsal
Session outcomes	Revised physical activity plan<br>Guidance to resources for self-monitoring of behaviour, e.g. exercise diary/log, smartphone application, wearable technology

Session 3

Session three (week 5) will focus on 'enabling' participants through *review of behaviour goals*, *problem solving* and *action planning*. The 'modelling' techniques from Session 2 may also be utilised. The physical activity plan should be reviewed

and adjusted as necessary to ensure a graded, progressive approach is taken across the intervention.

Session number & time point	Suggested Content, including behaviour change techniques
Session 3<br>Week 5	***Enablement techniques***<br>1.5 reviewing behaviour goals<br>1.2 problem solving and 1.4 action planning<br>***Modeling techniques***<br>2.2 feedback on behaviour<br>2.3 self-monitoring of behaviour<br>6.1 demonstration of behaviour<br>6.2 social comparison<br>8.1 behavioural practice/rehearsal
Session outcomes	Revised physical activity plan<br>Guidance to resources for self-monitoring of behaviour, e.g. exercise diary/log, smartphone application, wearable technology

Session 4

The **final (fourth) session,** (week 7) will focus on 'modelling' as per session two and three. Session four will also focus on preparing participants for longer term physical activity behaviour maintenance through a recapping on 'education' and 'enablement' techniques.

Session number & time point	Suggested Content, including behaviour change techniques
Session 4<br>Week 7	***Modeling techniques***<br>2.2 feedback on behaviour<br>2.3 self-monitoring of behaviour<br>6.1 demonstration of behaviour<br>6.2 social comparison<br>8.1 behavioural practice/rehearsal
Session outcomes	Longer term physical activity behaviour maintenance plan through recapping 'education' and 'enablement' techniques

Intervention detail log sheet

**Date:**<br><br>**Start Time:** **Finish time:**	**Session No:**
**Participant Name:**	**Physiotherapist:**
**Physical activity prescription:**<br><br>**Aerobic** ☐<br>**Strengthening** ☐	 <br>**Flexibility** ☐<br>**Balance/Proprioceptive** ☐
**Physical activity prescription details (Record F.I.T.T. details)**	
**Behaviour Change Techniques details (see BCT Taxonomy in Appendix)**	
**Adverse event(s)**<br>**Yes** ☐<br>**No** ☐	**Adverse event(s) details**
**Additional information**	

Delivering the intervention

Motivational interviewing (MI) is a counselling method that helps people resolve ambivalent feelings and insecurities to find the internal motivation they need to change their behaviour. MI is a collaborative, goal-oriented style of communication with a particular attention to the language of change. A commonly used definition of MI is: 'A directive, patient-centred counselling style for eliciting behaviour change by helping patients to explore and resolve ambivalence.' (Rollnick and Miller 1995). It is designed to strengthen personal motivation for and commitment to a specific goal by eliciting and exploring the person's own reasons for change within an atmosphere of acceptance and compassion (Miller and Rollnick 2012).

The five principles of MI are:

1. Express empathy through reflective listening
2. Develop discrepancy between clients' goals or values and their current behaviour
3. Avoid argument and direct confrontation
4. Adjust to client resistance rather than opposing it directly
5. Support self-efficacy and optimism

https://www.ncbi.nlm.nih.gov/books/NBK64964/

The core communication skills in MI are **OARS**:

- **O**pen questions
- **A**ffirming
- **R**eflecting
- **S**ummarising

The additional skill of giving information and advice with permission from the participant may also feature.

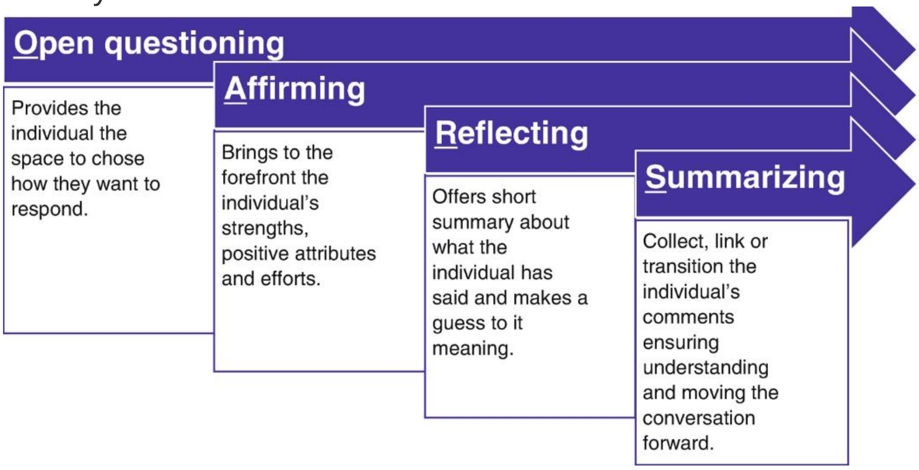

https://link.springer.com/chapter/10.1007/978-3-319-74742-2_6

Supporting physical activity – resources

Websites

Get Active Ireland https://www.getirelandactive.ie/

Arthritis Ireland https://www.arthritisireland.ie/

American College of Rheumatology https://www.rheumatology.org/I-Am-A/Patient-Caregiver/Diseases-Conditions/Living-Well-with-Rheumatic-Disease/Exercise-and-Arthritis

Moving Medicine UK https://movingmedicine.ac.uk/disease/inflammatory-rheumatic-disease-2/#start

Apps

- My Rheumatoid Arthritis (RA) Manager
- TRACK + REACT
- MyRA
- Rheumatoid Arthritis Diary
- Walk with Ease – 6 week walking programme
- Runkeeper
- Map My Run

Appendices

Appendix 1: Behaviour Change Taxonomy (v1) Michie et al (2013)

No.	Label	Definition	Examples
1. Goals and planning			
1.1	*Goal setting (behavior)*	Set or agree on a goal defined in terms of the behavior to be achieved<br><br>*Note: only code goal-setting if there is sufficient evidence that goal set as part of intervention; if goal unspecified or a behavioral outcome, code 1.3, Goal setting (outcome); if the goal defines a specific context, frequency, duration or intensity for the behavior, also code 1.4, Action planning*	Agree on a daily walking goal (e.g. 3 miles) with the person and reach agreement about the goal<br><br>Set the goal of eating 5 pieces of fruit per day as specified in public health guidelines
1.2	*Problem solving*	Analyse , or prompt the person to analyse, factors influencing the behavior and generate or select strategies that include overcoming barriers and/or increasing facilitators (includes 'Relapse Prevention' and 'Coping Planning')<br><br>*Note: barrier identification without solutions is not sufficient. If the BCT does not include analysing the behavioral problem, consider 12.3, Avoidance/changing exposure to cues for the behavior, 12.1, Restructuring the physical environment, 12.2, Restructuring the social environment, or 11.2, Reduce negative emotions*	Identify specific triggers (e.g. being in a pub, feeling anxious) that generate the urge/want/need to drink and develop strategies for avoiding environmental triggers or for managing negative emotions, such as anxiety, that motivate drinking<br><br>Prompt the patient to identify barriers preventing them from starting a new exercise regime e.g., lack of motivation, and discuss ways in which they could help overcome them e.g.,

			going to the gym with a buddy
1.3	*Goal setting (outcome)*	Set or agree on a goal defined in terms of a positive outcome of wanted behavior<br><br>*Note: only code guidelines if set as a goal in an intervention context; if goal is a behavior, code 1.1, Goal setting (behavior); if goal unspecified code 1.3, Goal setting (outcome)*	Set a weight loss goal (e.g. 0.5 kilogram over one week) as an outcome of changed eating patterns
1.4	*Action planning*	Prompt detailed planning of performance of the behavior (must include at least one of context, frequency, duration and intensity). Context may be environmental (physical or social) or internal (physical, emotional or cognitive) (includes *'*Implementation Intentions*'*)<br><br>*Note: evidence of action planning does not necessarily imply goal setting, only code latter if sufficient evidence*	Encourage a plan to carry condoms when going out socially at weekends<br><br>Prompt planning the performance of a particular physical activity (e.g. running) at a particular time (e.g. before work) on certain days of the week
1.5	*Review behavior goal(s)*	Review behavior goal(s) jointly with the person and consider modifying goal(s) or behavior change strategy in light of achievement. This may lead to re-setting the same goal, a small change in that goal or setting a new goal instead of (or in addition to) the first, or no change<br>*Note: if goal specified in terms of behavior, code 1.5, Review behavior goal(s), if goal unspecified, code 1.7, Review outcome goal(s); if*	Examine how well a person's performance corresponds to agreed goals e.g. whether they consumed less than one unit of alcohol per day, and consider modifying future behavioral goals accordingly e.g. by increasing or decreasing alcohol target or changing type of alcohol consumed

		discrepancy created consider also 1.6, Discrepancy between current behavior and goal	
1.6	*Discrepancy between current behavior and goal*	Draw attention to discrepancies between a person's current behavior (in terms of the *form, frequency, duration, or intensity* of that behavior) and the person's previously set outcome goals, behavioral goals or action plans (goes beyond self-monitoring of behavior)<br><br>*Note: if discomfort is created only code 13.3, Incompatible beliefs and not 1.6, Discrepancy between current behavior and goal; if goals are modified, also code 1.5, Review behavior goal(s) and/or 1.7, Review outcome goal(s); if feedback is provided, also code 2.2, Feedback on behaviour*	Point out that the recorded exercise fell short of the goal set
1.7	*Review outcome goal(s)*	Review outcome goal(s) jointly with the person and consider modifying goal(s) in light of achievement. This may lead to re-setting the same goal, a small change in that goal or setting a new goal instead of, or in addition to the first<br><br>*Note: if goal specified in terms of behavior, code 1.5, Review behavior goal(s), if goal unspecified, code 1.7, Review outcome goal(s); if discrepancy created consider also 1.6, Discrepancy between current behavior and goal*	Examine how much weight has been lost and consider modifying outcome goal(s) accordingly e.g., by increasing or decreasing subsequent weight loss targets

1.8	*Behavioral contract*	Create a written specification of the behavior to be performed, agreed on by the person, and witnessed by another<br><br>*Note: also code 1.1, Goal setting (behavior)*	Sign a contract with the person e.g. specifying that they will not drink alcohol for one week
1.9	*Commitment*	Ask the person to affirm or reaffirm statements indicating commitment to change the behavior<br><br>*Note: if defined in terms of the behavior to be achieved also code 1.1, Goal setting (behavior)*	Ask the person to use an "I will" statement to affirm or reaffirm a strong commitment (i.e. using the words "strongly", "committed" or "high priority") to start, continue or restart the attempt to take medication as prescribed
2. Feedback and monitoring			
2.1	*Monitoring of behavior by others without feedback*	Observe or record behavior with the person's knowledge as part of a behavior change strategy<br><br>*Note: if monitoring is part of a data collection procedure rather than a strategy aimed at changing behavior, do not code; if feedback given, code only 2.2, Feedback on behavior, and not 2.1, Monitoring of behavior by others without feedback; if monitoring outcome(s) code 2.5, Monitoring outcome(s) of behavior by others without feedback; if self-monitoring behavior, code 2.3, Self-monitoring of behaviour*	Watch hand washing behaviors among health care staff and make notes on context, frequency and technique used
2.2	*Feedback on behavior*	Monitor and provide informative or evaluative feedback on	Inform the person of how many steps they walked each day (as recorded on a

		performance of the behavior *(e.g. form, frequency, duration, intensity)*<br><br>*Note: if Biofeedback, code only 2.6, Biofeedback and not 2.2, Feedback on behavior; if feedback is on outcome(s) of behavior, code 2.7, Feedback on outcome(s) of behavior; if there is no clear evidence that feedback was given, code 2.1, Monitoring of behavior by others without feedback; if feedback on behaviour is evaluative e.g. praise, also code 10.4, Social reward*	pedometer) or how many calories they ate each day (based on a food consumption questionnaire).
2.3	*Self-monitoring of behavior*	Establish a method for the person to monitor and record their behavior(s) as part of a behavior change strategy<br>*Note: if monitoring is part of a data collection procedure rather than a strategy aimed at changing behavior, do not code; if monitoring of outcome of behavior, code 2.4, Self-monitoring of outcome(s) of behavior; if monitoring is by someone else (without feedback), code 2.1, Monitoring of behavior by others without feedback*	Ask the person to record daily, in a diary, whether they have brushed their teeth for at least two minutes before going to bed<br><br>Give patient a pedometer and a form for recording daily total number of steps
2.4	*Self-monitoring of outcome(s) of behavior*	Establish a method for the person to monitor and record the outcome(s) of their behavior as part of a behavior change strategy<br><br>*Note: if monitoring is part of a data collection procedure rather than a strategy aimed at changing behavior, do not code ; if monitoring behavior, code 2.3, Self-monitoring of behavior; if monitoring is by someone else*	Ask the person to weigh themselves at the end of each day, over a two week period, and record their daily weight on a graph to increase exercise behaviors

		(without feedback), code 2.5, Monitoring outcome(s) of behavior by others without feedback	
2.5	*Monitoring outcome(s) of behavior by others without feedback*	Observe or record outcomes of behavior with the person's knowledge as part of a behavior change strategy<br><br>*Note: if monitoring is part of a data collection procedure rather than a strategy aimed at changing behavior, do not code; if feedback given, code only 2.7, Feedback on outcome(s) of behavior; if monitoring behavior code 2.1, Monitoring of behavior by others without feedback; if self-monitoring outcome(s), code 2.4, Self-monitoring of outcome(s) of behavior*	Record blood pressure, blood glucose, weight loss, or physical fitness
2.6	*Biofeedback*	Provide feedback about the body *(e.g. physiological or biochemical state)* using an external monitoring device as part of a behavior change strategy<br><br>*Note: if Biofeedback, code only 2.6, Biofeedback and <u>not</u> 2.2, Feedback on behavior or 2.7, Feedback on outcome(s) of behaviour*	Inform the person of their blood pressure reading to improve adoption of health behaviors
2.7	*Feedback on outcome(s) of behavior*	Monitor and provide feedback on the outcome of performance of the behavior<br><br>*Note: if Biofeedback, code only 2.6, Biofeedback and <u>not</u> 2.7, Feedback on outcome(s) of behavior; if feedback is on behavior code 2.2, Feedback on behavior; if there is no*	Inform the person of how much weight they have lost following the implementation of a new exercise regime

		clear evidence that feedback was given code 2.5, Monitoring outcome(s) of behavior by others without feedback; if feedback on behaviour is evaluative e.g. praise, also code 10.4, Social reward	
3. Social support			
3.1	*Social support (unspecified)*	Advise on, arrange or provide social support *(e.g. from friends, relatives, colleagues,' buddies' or staff)* or non-contingent praise or reward for performance of the behavior*.* It includes encouragement and counselling, but only when it is directed at the behavior<br><br>*Note: attending a group class and/or mention of 'follow-up' does not necessarily apply this BCT, support must be explicitly mentioned; if practical, code 3.2, Social support (practical); if emotional, code 3.3, Social support (emotional)* (includes 'Motivational interviewing' and 'Cognitive Behavioral Therapy')	Advise the person to call a 'buddy' when they experience an urge to smoke<br><br>Arrange for a housemate to encourage continuation with the behavior change programme<br><br>Give information about a self-help group that offers support for the behavior
3.2	*Social support (practical)*	Advise on, arrange, or provide practical help *(e.g. from friends, relatives, colleagues, 'buddies' or staff)* for performance of the behavior<br><br>*Note: if emotional, code 3.3, Social support (emotional); if general or unspecified, code 3.1, Social support (unspecified) If only restructuring the physical environment or adding objects to the environment, code 12.1, Restructuring the physical environment or 12.5, Adding objects*	Ask the partner of the patient to put their tablet on the breakfast tray so that the patient remembers to take it

		to the environment; attending a group or class and/or mention of 'follow-up' does not necessarily apply this BCT, support must be explicitly mentioned.	
3.3	*Social support (emotional)*	Advise on, arrange, or provide emotional social support *(e.g. from friends, relatives, colleagues, 'buddies' or staff)* for performance of the behavior<br><br>*Note: if practical, code 3.2, Social support (practical); if unspecified, code 3.1, Social support (unspecified)*	Ask the patient to take a partner or friend with them to their colonoscopy appointment
4. Shaping knowledge			
4.1	*Instruction on how to perform a behavior*	Advise or agree on how to perform the behavior (includes '<u>Skills training</u>')<br><br>*Note: when the person attends classes such as exercise or cookery, code 4.1, Instruction on how to perform the behavior, 8.1, Behavioral practice/rehearsal <u>and</u> 6.1, Demonstration of the behavior*	Advise the person how to put a condom on a model of a penis correctly
4.2	*Information about antecedents*	Provide information about antecedents<br><br>(*e.g. social and environmental situations and events, emotions, cognitions)* that reliably predict performance of the behaviour	Advise to keep a record of snacking and of situations or events occurring prior to snacking
4.3	*Re-attribution*	Elicit perceived causes of behavior and suggest alternative	If the person attributes their over-eating to the frequent presence of delicious food,

		explanations *(e.g. external or internal and stable or unstable)*	suggest that the 'real' cause may be the person's inattention to bodily signals of hunger and satiety
4.4	*Behavioral experiments*	Advise on how to identify and test hypotheses about the behavior, its causes and consequences, by collecting and interpreting data	Ask a family physician to give evidence-based advice rather than prescribe antibiotics and to note whether the patients are grateful or annoyed
5. Natural consequences			
5.1	*Information about health consequences*	Provide information (e.g. written, verbal, visual) about health consequences of performing the behavior<br><br>*Note: consequences can be for any target, not just the recipient(s) of the intervention; emphasising importance of consequences is not sufficient; if information about emotional consequences, code 5.6, Information about emotional consequences; if about social, environmental or unspecified consequences code 5.3, Information about social and environmental consequences*	Explain that not finishing a course of antibiotics can increase susceptibility to future infection<br><br>Present the likelihood of contracting a sexually transmitted infection following unprotected sexual behavior
5.2	*Salience of consequences*	Use methods specifically designed to emphasise the consequences of performing the behaviour with the aim of making them more memorable (goes beyond informing about consequences)<br><br>*Note: if information about consequences, also code 5.1, Information about health*	Produce cigarette packets showing pictures of health consequences e.g. diseased lungs, to highlight the dangers of continuing to smoke

		consequences, 5.6, Information about emotional consequences or 5.3, Information about social and environmental consequences	
5.3	*Information about social and environmental consequences*	Provide information (e.g. written, verbal, visual) about social and environmental consequences of performing the behavior<br><br>*Note: consequences can be for any target, not just the recipient(s) of the intervention; if information about health or consequences, code 5.1, Information about health consequences; if about emotional consequences, code 5.6, Information about emotional consequences; if unspecified, code 5.3, Information about social and environmental consequences*	Tell family physician about financial remuneration for conducting health screening<br><br>Inform a smoker that the majority of people disapprove of smoking in public places
5.4	*Monitoring of emotional consequences*	Prompt assessment of feelings after attempts at performing the behavior	Agree that the person will record how they feel after taking their daily walk
5.5	*Anticipated regret*	Induce or raise awareness of expectations of future regret about performance of the unwanted behavior<br><br>*Note: not including 5.6, Information about emotional consequences; if suggests adoption of a perspective or new perspective in order to change cognitions also code 13.2, Framing/reframing*	Ask the person to assess the degree of regret they will feel if they do not quit smoking

5.6	*Information about emotional consequences*	Provide information (e.g. written, verbal, visual) about emotional consequences of performing the behavior<br><br>*Note: consequences can be related to emotional health disorders (e.g. depression, anxiety) and/or states of mind (e.g. low mood, stress); not including 5.5, Anticipated regret; consequences can be for any target, not just the recipient(s) of the intervention; if information about health consequences code 5.1, Information about health consequences; if about social, environmental or unspecified code 5.3, Information about social and environmental consequences*	Explain that quitting smoking increases happiness and life satisfaction
6. Comparison of behaviour			
6.1	*Demonstration of the behavior*	Provide an observable sample of the performance of the behaviour, directly in person or indirectly e.g. via film, pictures, for the person to aspire to or imitate (includes 'Modelling'). *Note:* if advised to practice, also code, *8.1, Behavioural practice and rehearsal; If provided with instructions on how to perform, also code 4.1, Instruction on how to perform the behaviour*	Demonstrate to nurses how to raise the issue of excessive drinking with patients via a role-play exercise
6.2	*Social comparison*	Draw attention to others' performance to allow comparison with the person's own performance *Note: being in a group setting does not necessarily mean that social comparison is actually taking place*	Show the doctor the proportion of patients who were prescribed antibiotics for a common cold by other doctors and compare with their own data

6.3	*Information about others' approval*	Provide information about what other people think about the behavior. The information clarifies whether others will like, approve or disapprove of what the person is doing or will do	Tell the staff at the hospital ward that staff at all other wards approve of washing their hands according to the guidelines
7. Associations			
7.1	*Prompts/cues*	Introduce or define environmental or social stimulus with the purpose of prompting or cueing the behavior. The prompt or cue would normally occur at the time or place of performance<br><br>*Note: when a stimulus is linked to a specific action in an if-then plan including one or more of frequency, duration or intensity also code 1.4, Action planning.*	Put a sticker on the bathroom mirror to remind people to brush their teeth
7.2	*Cue signalling reward*	Identify an environmental stimulus that reliably predicts that reward will follow the behavior (includes 'Discriminative cue')	Advise that a fee will be paid to dentists for a particular dental treatment of 6-8 year old, but not older, children to encourage delivery of that treatment (the 6-8 year old children are the environmental stimulus)

7.3	*Reduce prompts/cues*	Withdraw gradually prompts to perform the behavior (includes 'Fading')	Reduce gradually the number of reminders used to take medication
7.4	*Remove access to the reward*	Advise or arrange for the person to be separated from situations in which unwanted behavior can be rewarded in order to reduce the behavior (includes 'Time out')	Arrange for cupboard containing high calorie snacks to be locked for a specified period to reduce the consumption of sugary foods in between meals
7.5	*Remove aversive stimulus*	Advise or arrange for the removal of an aversive stimulus to facilitate behavior change (includes 'Escape learning')	Arrange for a gym-buddy to stop nagging the person to do more exercise in order to increase the desired exercise behaviour
7.6	*Satiation*	Advise or arrange repeated exposure to a stimulus that reduces or extinguishes a drive for the unwanted behavior	Arrange for the person to eat large quantities of chocolate, in order to reduce the person's appetite for sweet foods
7.7	*Exposure*	Provide systematic confrontation with a feared stimulus to reduce the response to a later encounter	Agree a schedule by which the person who is frightened of surgery will visit the hospital where they are scheduled to have surgery
7.8	*Associative learning*	Present a neutral stimulus jointly with a stimulus that already elicits the behavior repeatedly until the neutral stimulus elicits that behavior (includes 'Classical/Pavlovian Conditioning')	Present repeatedly fatty foods with a disliked sauce to discourage the consumption of fatty foods

		Note: when a BCT involves reward or punishment, code one or more of: 10.2, Material reward (behavior); 10.3, Non-specific reward; 10.4, Social reward, 10.9, Self-reward; 10.10, Reward (outcome)	
8. Repetition and substitution			
8.1	*Behavioral practice/ rehearsal*	Prompt practice or rehearsal of the performance of the behavior one or more times in a context or at a time when the performance may not be necessary, in order to increase habit and skill<br><br>*Note: if aiming to associate performance with the context, <u>also</u> code 8.3, Habit formation*	Prompt asthma patients to practice measuring their peak flow in the nurse's consulting room
8.2	*Behavior substitution*	Prompt substitution of the unwanted behavior with a wanted or neutral behavior<br><br>*Note: if this occurs regularly, <u>also</u> code 8.4, Habit reversal*	Suggest that the person goes for a walk rather than watches television
8.3	*Habit formation*	Prompt rehearsal and repetition of the behavior in the same context repeatedly so that the context elicits the behavior<br><br>*Note: <u>also</u> code 8.1, Behavioral practice/rehearsal*	Prompt patients to take their statin tablet before brushing their teeth every evening
8.4	*Habit reversal*	Prompt rehearsal and repetition of an alternative behavior to replace an unwanted habitual behavior	Ask the person to walk up stairs at work where they previously always took the lift

		Note: also code 8.2, Behavior substitution	
8.5	*Overcorrection*	Ask to repeat the wanted behavior in an exaggerated way following an unwanted behaviour	Ask to eat only fruit and vegetables the day after a poor diet
8.6	*Generalisation of a target behavior*	Advise to perform the wanted behaviour, which is already performed in a particular situation, in another situation	Advise to repeat toning exercises learned in the gym when at home
8.7	*Graded tasks*	Set easy-to-perform tasks, making them increasingly difficult, but achievable, until behavior is performed	Ask the person to walk for 100 yards a day for the first week, then half a mile a day after they have successfully achieved 100 yards, then two miles a day after they have successfully achieved one mile
9. Comparison of outcomes			
9.1	*Credible source*	Present verbal or visual communication from a credible source in favour of or against the behavior<br><br>*Note: code this BCT if source generally agreed on as credible e.g., health professionals, celebrities or words used to indicate expertise or leader in field and if the communication has the aim of persuading; if information about health consequences, also code 5.1, Information about health consequences, if about emotional consequences, also code 5.6, Information about emotional*	Present a speech given by a high status professional to emphasise the importance of not exposing patients to unnecessary radiation by ordering x-rays for back pain

		consequences; if about social, environmental or unspecified consequences also code 5.3, Information about social and environmental consequences	
9.2	*Pros and cons*	Advise the person to identify and compare reasons for wanting (pros) and not wanting to (cons) change the behavior (includes 'Decisional balance'*)*<br><br>*Note: if providing information about health consequences, also code 5.1, Information about health consequences; if providing information about emotional consequences, also code 5.6, Information about emotional consequences; if providing information about social, environmental or unspecified consequences also code 5.3, Information about social and environmental consequences*	Advise the person to list and compare the advantages and disadvantages of prescribing antibiotics for upper respiratory tract infections
9.3	*Comparative imagining of future outcomes*	Prompt or advise the imagining and comparing of future outcomes of changed versus unchanged behaviour	Prompt the person to imagine and compare likely or possible outcomes following attending versus not attending a screening appointment
10. Reward and threat			

10.1	*Material incentive (behavior)*	Inform that money, vouchers or other valued objects *will be* delivered if and only if there has been effort and/or progress in performing the behavior (includes 'Positive reinforcement')<br><br>*Note: if incentive is social, code 10.5, Social incentive if unspecified code 10.6, Non-specific incentive, and not 10.1, Material incentive (behavior); if incentive is for outcome, code 10.8, Incentive (outcome). If reward is delivered also code one of: 10.2, Material reward (behavior); 10.3, Non-specific reward; 10.4, Social reward, 10.9, Self-reward; 10.10, Reward (outcome)*	Inform that a financial payment will be made each month in pregnancy that the woman has not smoked
10.2	*Material reward (behavior)*	Arrange for the delivery of money, vouchers or other valued objects if and only if there *has been* effort and/or progress in performing the behavior (includes 'Positive reinforcement')<br><br>*Note: If reward is social, code 10.4, Social reward, if unspecified code 10.3, Non-specific reward, and not 10.1, Material reward (behavior); if reward is for outcome, code 10.10, Reward (outcome). If informed of reward in advance of rewarded behaviour, also code one of: 10.1, Material incentive (behaviour); 10.5, Social incentive; 10.6, Non-specific incentive; 10.7, Self-incentive; 10.8, Incentive (outcome)*	Arrange for the person to receive money that would have been spent on cigarettes if and only if the smoker has not smoked for one month
10.3	*Non-specific reward*	Arrange delivery of a reward if and only if there *has been* effort and/or	Identify something (e.g. an activity such as a visit to the cinema) that the person

		progress in performing the behavior (includes 'Positive reinforcement')<br><br>*Note: if reward is material, code 10.2, Material reward (behavior), if social, code 10.4, Social reward, and not 10.3, Non-specific reward; if reward is for outcome code 10.10, Reward (outcome). If informed of reward in advance of rewarded behaviour, also code one of: 10.1, Material incentive (behaviour); 10.5, Social incentive; 10.6, Non-specific incentive; 10.7, Self-incentive; 10.8, Incentive (outcome)*	values and arrange for this to be delivered if and only if they attend for health screening
10.4	*Social reward*	Arrange verbal or non-verbal reward if and only if there *has been* effort and/or progress in performing the behavior (includes 'Positive reinforcement')<br><br>*Note: if reward is material, code 10.2, Material reward (behavior), if unspecified code 10.3, Non-specific reward, and not 10.4, Social reward; if reward is for outcome code 10.10, Reward (outcome). If informed of reward in advance of rewarded behaviour, also code one of: 10.1, Material incentive (behaviour); 10.5, Social incentive; 10.6, Non-specific incentive; 10.7, Self-incentive; 10.8, Incentive (outcome)*	Congratulate the person for each day they eat a reduced fat diet
10.5	*Social incentive*	Inform that a verbal or non-verbal reward *will be* delivered if and only if there has been effort and/or progress in performing the behavior (includes 'Positive reinforcement')	Inform that they will be congratulated for each day they eat a reduced fat diet

		Note: if incentive is material, code 10.1, Material incentive (behavior), if unspecified code 10.6, Non-specific incentive, and not 10.5, Social incentive; if incentive is for outcome code 10.8, Incentive (outcome). If reward is delivered also code one of: 10.2, Material reward (behavior); 10.3, Non-specific reward; 10.4, Social reward, 10.9, Self-reward; 10.10, Reward (outcome)	
10.6	*Non-specific incentive*	Inform that a reward *will be* delivered if and only if there has been effort and/or progress in performing the behavior (includes 'Positive reinforcement')<br><br>*Note: if incentive is material, code 10.1, Material incentive (behavior), if social, code 10.5, Social incentive and not 10.6, Non-specific incentive; if incentive is for outcome code 10.8, Incentive (outcome). If reward is delivered also code one of: 10.2, Material reward (behavior); 10.3, Non-specific reward; 10.4, Social reward, 10.9, Self-reward; 10.10, Reward (outcome)*	Identify an activity that the person values and inform them that this will happen if and only if they attend for health screening
10.7	*Self-incentive*	Plan to reward self in future if and only if there has been effort and/or progress in performing the behavior<br><br>*Note: if self-reward is material, also code 10.1, Material incentive (behavior), if social, also code 10.5, Social incentive, if unspecified, also code 10.6, Non-specific incentive; if incentive is for outcome code 10.8, Incentive (outcome). If reward is delivered also code one of: 10.2,*	Encourage to provide self with material (e.g., new clothes) or other valued objects if and only if they have adhered to a healthy diet

		Material reward (behavior); 10.3, Non-specific reward; 10.4, Social reward, 10.9, Self-reward; 10.10, Reward (outcome)	
10.8	*Incentive (outcome)*	Inform that a reward *will be* delivered if and only if there has been effort and/or progress in achieving the behavioural outcome (*includes '*Positive reinforcement*'*)<br><br>*Note: this includes social, material, self- and non-specific incentives for outcome; if incentive is for the behavior code 10.5, Social incentive, 10.1, Material incentive (behavior), 10.6, Non-specific incentive or 10.7, Self-incentive and* not *10.8, Incentive (outcome). If reward is delivered also code one of: 10.2, Material reward (behavior); 10.3, Non-specific reward; 10.4, Social reward, 10.9, Self-reward; 10.10, Reward (outcome)*	Inform the person that they will receive money if and only if a certain amount of weight is lost
10.9	*Self-reward*	Prompt self-praise or self-reward if and only if there *has been* effort and/or progress in performing the behavior<br><br>*Note: if self-reward is material,* also *code 10.2, Material reward (behavior), if social,* also *code 10.4, Social reward, if unspecified,* also *code 10.3, Non-specific reward; if reward is for outcome code 10.10, Reward (outcome). If informed of reward in advance of rewarded behaviour, also code one of: 10.1, Material incentive (behaviour); 10.5, Social incentive; 10.6, Non-*	Encourage to reward self with material (e.g., new clothes) or other valued objects if and only if they have adhered to a healthy diet

		specific incentive; 10.7, Self-incentive; 10.8, Incentive (outcome)	
10.10	*Reward (outcome)*	Arrange for the delivery of a reward if and only if there *has been* effort and/or progress in achieving the behavioral outcome (includes 'Positive reinforcement')<br><br>*Note: this includes social, material, self- and non-specific rewards for outcome; if reward is for the behavior code 10.4, Social reward, 10.2, Material reward (behavior), 10.3, Non-specific reward or 10.9, Self-reward and not 10.10, Reward (outcome). If informed of reward in advance of rewarded behaviour, also code one of: 10.1, Material incentive (behaviour); 10.5, Social incentive; 10.6, Non-specific incentive; 10.7, Self-incentive; 10.8, Incentive (outcome)*	Arrange for the person to receive money if and only if a certain amount of weight is lost
10.11	*Future punishment*	Inform that future punishment or removal of reward will be a consequence of performance of an unwanted behavior (may include fear arousal) (includes 'Threat')	Inform that continuing to consume 30 units of alcohol per day is likely to result in loss of employment if the person continues
11. Regulation			
11.1	*Pharmacological support*	Provide, or encourage the use of or adherence to, drugs to facilitate behavior change<br><br>*Note: if pharmacological support to reduce negative emotions (i.e. anxiety) then also code 11.2, Reduce negative emotions*	Suggest the patient asks the family physician for nicotine replacement therapy to facilitate smoking cessation

11.2	*Reduce negative emotions* [b]	Advise on ways of reducing negative emotions to facilitate performance of the behavior (includes 'Stress Management')<br><br>*Note: if includes analysing the behavioural problem, also code 1.2, Problem solving*	Advise on the use of stress management skills, e.g. to reduce anxiety about joining Alcoholics Anonymous
11.3	*Conserving mental resources*	Advise on ways of minimising demands on mental resources to facilitate behavior change	Advise to carry food calorie content information to reduce the burden on memory in making food choices
11.4	*Paradoxical instructions*	Advise to engage in some form of the unwanted behavior with the aim of reducing motivation to engage in that behaviour	Advise a smoker to smoke twice as many cigarettes a day as they usually do<br><br>Tell the person to stay awake as long as possible in order to reduce insomnia
12. Antecedents			
12.1	*Restructuring the physical environment*	Change, or advise to change the physical environment in order to facilitate performance of the wanted behavior or create barriers to the unwanted behavior (other than prompts/cues, rewards and punishments)<br><br>*Note: this may also involve 12.3, Avoidance/reducing exposure to cues for the behavior; if restructuring of the social*	Advise to keep biscuits and snacks in a cupboard that is inconvenient to get to<br><br>Arrange to move vending machine out of the school

		environment code 12.2, Restructuring the social environment;<br><br>*if only adding objects to the environment, code 12.5, Adding objects to the environment*	
12.2	*Restructuring the social environment*	Change, or advise to change the social environment in order to facilitate performance of the wanted behavior or create barriers to the unwanted behavior (other than prompts/cues, rewards and punishments)<br><br>*Note: this may also involve 12.3, Avoidance/reducing exposure to cues for the behavior; if also restructuring of the physical environment also code 12.1, Restructuring the physical environment*	Advise to minimise time spent with friends who drink heavily to reduce alcohol consumption
12.3	*Avoidance/reducing exposure to cues for the behavior*	Advise on how to avoid exposure to specific social and contextual/physical cues for the behavior, including changing daily or weekly routines<br><br>*Note: this may also involve 12.1, Restructuring the physical environment* and/or *12.2, Restructuring the social environment*; if the BCT includes analysing the behavioral problem, only code *1.2*, *Problem solving*	Suggest to a person who wants to quit smoking that their social life focus on activities other than pubs and bars which have been associated with smoking
12.4	*Distraction*	Advise or arrange to use an alternative focus for attention to	Suggest to a person who is trying to avoid between-meal snacking to focus on a topic they enjoy (e.g.

		avoid triggers for unwanted behaviour	holiday plans) instead of focusing on food
12.5	*Adding objects to the environment*	Add objects to the environment in order to facilitate performance of the behavior<br><br>*Note: Provision of information (e.g. written, verbal, visual) in a booklet or leaflet is insufficient. If this is accompanied by social support, also code 3.2, Social support (practical); if the environment is changed beyond the addition of objects, also code 12.1, Restructuring the physical environment*	Provide free condoms to facilitate safe sex<br><br>Provide attractive toothbrush to improve tooth brushing technique
12.6	*Body changes*	Alter body structure, functioning or support directly to facilitate behavior change	Prompt strength training, relaxation training or provide assistive aids (e.g. a hearing aid)
13. Identity			
13.1	*Identification of self as role model*	Inform that one's own behavior may be an example to others	Inform the person that if they eat healthily, that may be a good example for their children
13.2	*Framing/reframing*	Suggest the deliberate adoption of a perspective or new perspective on behavior (e.g. its purpose) in order to change cognitions or emotions about performing the behavior (includes 'Cognitive structuring'); *If information about consequences then code 5.1, Information about health consequences, 5.6, Information about emotional consequences or*	Suggest that the person might think of the tasks as reducing sedentary behavior (rather than increasing activity)

		5.3, Information about social and environmental consequences instead of 13.2, Framing/reframing	
13.3	*Incompatible beliefs*	Draw attention to discrepancies between current or past behavior and self-image, in order to create discomfort (includes 'Cognitive dissonance')	Draw attention to a doctor's liberal use of blood transfusion and their self-identification as a proponent of evidence-based medical practice
13.4	*Valued self-identity*	Advise the person to write or complete rating scales about a cherished value or personal strength as a means of affirming the person's identity as part of a behavior change strategy (includes 'Self-affirmation')	Advise the person to write about their personal strengths before they receive a message advocating the behavior change
13.5	*Identity associated with changed behavior*	Advise the person to construct a new self-identity as someone who 'used to engage with the unwanted behavior'	Ask the person to articulate their new identity as an 'ex-smoker'
14. Scheduled consequences			
14.1	*Behavior cost*	Arrange for withdrawal of something valued if and only if an unwanted behavior is performed (includes 'Response cost'). Note if withdrawal of contingent reward code*, 14.3, Remove reward*	Subtract money from a prepaid refundable deposit when a cigarette is smoked

14.2	*Punishment*	Arrange for aversive consequence contingent on the performance of the unwanted behavior	Arrange for the person to wear unattractive clothes following consumption of fatty foods
14.3	*Remove reward*	Arrange for discontinuation of contingent reward following performance of the unwanted behavior (includes 'Extinction')	Arrange for the other people in the household to ignore the person every time they eat chocolate (rather than attending to them by criticising or persuading)
14.4	*Reward approximation*	Arrange for reward following any approximation to the target behavior, gradually rewarding only performance closer to the wanted behavior (includes 'Shaping')<br><br>*Note: also code one of 59-63*	Arrange reward for any reduction in daily calories, gradually requiring the daily calorie count to become closer to the planned calorie intake
14.5	*Rewarding completion*	Build up behavior by arranging reward following final component of the behavior; gradually add the components of the behavior that occur earlier in the behavioral sequence (includes 'Backward chaining')<br><br>*Note: also code one of 10.2, Material reward (behavior); 10.3, Non-specific reward; 10.4, Social reward, 10.9, Self-reward; 10.10, Reward (outcome)*	Reward eating a supplied low calorie meal; then make reward contingent on cooking and eating the meal; then make reward contingent on purchasing, cooking and eating the meal
14.6	*Situation-specific reward*	Arrange for reward following the behavior in one situation but not in another (includes 'Discrimination training')	Arrange reward for eating at mealtimes but not between meals

		Note: also code one of 10.2, Material reward (behavior); 10.3, Non-specific reward; 10.4, Social reward, 10.9, Self-reward; 10.10, Reward (outcome)	
14.7	*Reward incompatible behavior*	Arrange reward for responding in a manner that is incompatible with a previous response to that situation (includes 'Counter-conditioning')<br><br>*Note: also code one of 10.2, Material reward (behavior); 10.3, Non-specific reward; 10.4, Social reward, 10.9, Self-reward; 10.10, Reward (outcome)*	Arrange reward for ordering a soft drink at the bar rather than an alcoholic beverage
14.8	*Reward alternative behavior*	Arrange reward for performance of an alternative to the unwanted behavior (includes 'Differential reinforcement')<br><br>*Note: also code one of 10.2, Material reward (behavior); 10.3, Non-specific reward; 10.4, Social reward, 10.9, Self-reward; 10.10, Reward (outcome); consider also coding 1.2, Problem solving*	Reward for consumption of low fat foods but not consumption of high fat foods
14.9	*Reduce reward frequency*	Arrange for rewards to be made contingent on increasing duration or frequency of the behavior (includes 'Thinning')<br><br>*Note: also code one of 10.2, Material reward (behavior); 10.3, Non-specific reward; 10.4, Social reward, 10.9, Self-reward; 10.10, Reward (outcome)*	Arrange reward for each day without smoking, then each week, then each month, then every 2 months and so on

14.10	*Remove punishment*	Arrange for removal of an unpleasant consequence contingent on performance of the wanted behavior (includes 'Negative reinforcement')	Arrange for someone else to do housecleaning only if the person has adhered to the medication regimen for a week
15. Self-belief			
15.1	*Verbal persuasion about capability*	Tell the person that they can successfully perform the wanted behavior, arguing against self-doubts and asserting that they can and will succeed	Tell the person that they can successfully increase their physical activity, despite their recent heart attack.
15.2	*Mental rehearsal of successful performance*	Advise to practise imagining performing the behavior successfully in relevant contexts	Advise to imagine eating and enjoying a salad in a work canteen
15.3	*Focus on past success*	Advise to think about or list previous successes in performing the behavior (or parts of it)	Advise to describe or list the occasions on which the person had ordered a non-alcoholic drink in a bar
15.4	*Self-talk*	Prompt positive self-talk (aloud or silently) before and during the behavior	Prompt the person to tell themselves that a walk will be energising
16. Covert learning			
16.1	*Imaginary punishment*	Advise to imagine performing the unwanted behavior in a real-life situation followed by imagining an unpleasant consequence (includes 'Covert sensitisation')	Advise to imagine overeating and then vomiting
16.2	*Imaginary reward*	Advise to imagine performing the wanted behavior in a real-life	Advise the health professional to imagine

		situation followed by imagining a pleasant consequence (includes 'Covert conditioning')	giving dietary advice followed by the patient losing weight and no longer being diabetic
16.3	*Vicarious consequences*	Prompt observation of the consequences (including rewards and punishments) for others when they perform the behavior<br><br>*Note: if observation of health consequences, also code 5.1, Information about health consequences; if of emotional consequences, also code 5.6, Information about emotional consequences, if of social, environmental or unspecified consequences, also code 5.3, Information about social and environmental consequences*	Draw attention to the positive comments other staff get when they disinfect their hands regularly

Appendix 2: Behaviour Change Taxonomy (v1) Michie et al (2013) – short version

Page	Grouping and BCTs
1	**1. Goals and planning**
	1.1. Goal setting (behavior)
	1.2. Problem solving
	1.3. Goal setting (outcome)
	1.4. Action planning
	1.5. Review behavior goal(s)
	1.6. Discrepancy between current behavior and goal
	1.7. Review outcome goal(s)
	1.8. Behavioral contract
	1.9. Commitment
3	**2. Feedback and monitoring**
	2.1. Monitoring of behavior by others without feedback
	2.2. Feedback on behaviour
	2.3. Self-monitoring of behaviour
	2.4. Self-monitoring of outcome(s) of behaviour
	2.5. Monitoring of outcome(s) of behavior without feedback
	2.6. Biofeedback
	2.7. Feedback on outcome(s) of behavior
5	**3. Social support**
	3.1. Social support (unspecified)
	3.2. Social support (practical)
	3.3. Social support (emotional)
6	**4. Shaping knowledge**
	4.1. Instruction on how to perform the behavior
	4.2. Information about Antecedents
	4.3. Re-attribution
	4.4. Behavioral experiments
7	**5. Natural consequences**
	5.1. Information about health consequences
	5.2. Salience of consequences
	5.3. Information about social and environmental consequences
	5.4. Monitoring of emotional consequences
	5.5. Anticipated regret
	5.6. Information about emotional consequences

Page	Grouping and BCTs
8	**6. Comparison of behaviour**
	6.1. Demonstration of the behavior
	6.2. Social comparison
	6.3. Information about others' approval
9	**7. Associations**
	7.1. Prompts/cues
	7.2. Cue signalling reward
	7.3. Reduce prompts/cues
	7.4. Remove access to the reward
	7.5. Remove aversive stimulus
	7.6. Satiation
	7.7. Exposure
	7.8. Associative learning
10	**8. Repetition and substitution**
	8.1. Behavioral practice/rehearsal
	8.2. Behavior substitution
	8.3. Habit formation
	8.4. Habit reversal
	8.5. Overcorrection
	8.6. Generalisation of target behavior
	8.7. Graded tasks
11	**9. Comparison of outcomes**
	9.1. Credible source
	9.2. Pros and cons
	9.3. Comparative imagining of future outcomes
12	**10. Reward and threat**
	10.1. Material incentive (behavior)
	10.2. Material reward (behavior)
	10.3. Non-specific reward
	10.4. Social reward
	10.5. Social incentive
	10.6. Non-specific incentive
	10.7. Self-incentive
	10.8. Incentive (outcome)
	10.9. Self-reward
	10.10. Reward (outcome)
	10.11. Future punishment
15	**11. Regulation**
	11.1. Pharmacological support
	11.2. Reduce negative emotions
	11.3. Conserving mental resources
	11.4. Paradoxical instructions

Page	Grouping and BCTs
16	**12. Antecedents**
	12.1. Restructuring the physical environment
	12.2. Restructuring the social environment
	12.3. Avoidance/reducing exposure to cues for the behavior
	12.4. Distraction
	12.5. Adding objects to the environment
	12.6. Body changes
17	**13. Identity**
	13.1. Identification of self as role model
	13.2. Framing/reframing
	13.3. Incompatible beliefs
	13.4. Valued self-identify
	13.5. Identity associated with changed behavior
18	**14. Scheduled consequences**
	14.1. Behavior cost
	14.2. Punishment
	14.3. Remove reward
	14.4. Reward approximation
	14.5. Rewarding completion
	14.6. Situation-specific reward
	14.7. Reward incompatible behavior
	14.8. Reward alternative behavior
	14.9. Reduce reward frequency
	14.10. Remove punishment
19	**15. Self-belief**
	15.1. Verbal persuasion about capability
	15.2. Mental rehearsal of successful performance
	15.3. Focus on past success
	15.4. Self-talk
19	**16. Covert learning**
	16.1. Imaginary punishment
	16.2. Imaginary reward
	16.3. Vicarious consequences
