# Supplementary material for: Promoting physical activity in rheumatoid arthritis through a physiotherapist led behaviour change-based intervention (PIPPRA): a feasibility randomised trial
Source: Rheumatol Int. 2024 Mar 4;44(5):779–93. doi: 10.1007/s00296-024-05544-1 (PMC10980645; doi:10.1007/s00296-024-05544-1)
Supplement: Supplementary file 2 — Supplementary file2 (PDF 329 KB) [file 296_2024_5544_MOESM2_ESM.pdf]

PIPPRA RESEARCH STUDY INFORMATION

The PIPPRA will examine physical activity in people with rheumatoid arthritis (RA). The aim of the study is to work out the practicalities of doing the study before we proceed to do it as a larger study in different rheumatology centres in Ireland. This first step is important to see how the programme works, how easy it is to recruit people with RA to take part and to see what participants think of the intervention and the assessment measures used.

The PIPPRA project partners include:

Arthritis Ireland
Little Things make a Big Difference

Ospidéil OL
UL Hospitals
Working together, caring for you

ISCP
Chartered Physiotherapists in Rheumatology

Irish Society for Rheumatology

Contact Details

Phone: 086-8517636

E-mail:
PIPPRA
Postdoctoral Researcher

Dr. Louise Larkin

Louise.Larkin@ul.ie

Health Research Institute
Clinical Education and Research Centre
University Hospital Limerick
Dooradoyle
Limerick

PIPPRA

Physical Activity Information Leaflet

PIPPRA

Physiotherapist led Intervention to Promote Physical activity in Rheumatoid Arthritis - a pilot study

PIPPRA Project

The **PIPPRA** project is part of Arthritis Research Limerick, a group of researchers and clinicians based at the University of Limerick and University Hospitals Limerick. The PIPPRA project is funded by the ***Health Research Board***.

PHYSICAL ACTIVITY

Physical activity is an important part in managing your arthritis and is proven to have many benefits in managing arthritis. This includes helping to managing your arthritis symptoms, such as pain, and improving your mental and physical health. Being physically active can improve your joint movement, muscle strength and heart health. It can also help with weight management, which reduces your risk of other health conditions, such as diabetes or stroke.

Physical activity is any bodily movement that uses energy. Examples include climbing the stairs, cleaning, walking in shops and lots of other daily activities.

Exercise is a subset of physical activity. It's a type of physical activity that is planned, structured and is for the purpose of improving your physical fitness.

Physical Activity Recommendations

The European League Against Rheumatism 2018 guidelines for people who have rheumatoid arthritis advise the following:

Cardiorespiratory/Aerobic Exercise

Moderate intensity aerobic physical activity for at least 30 minutes on 5 days per week

or

vigorous intensity aerobic activity for a minimum of 20 minutes on 3 days per week.

Talk test

The talk test can help to figure out how what intensity you are exercising at. At **low intensity** you can talk and sing a song, at **moderate intensity** you can have a conversation but can't sing a song, whereas when exercising at a **vigorous intensity** you can't have a conversation.

Strength/Resistance Exercise

Strength exercises for each major muscle group should be trained on 2–3 days per week . Major muscle groups include the thigh muscles, buttock muscles, and arm muscles. A variety of exercise equipment and/or body weight can be used to perform these exercises.

Flexibility Exercise

Flexibility exercises includes stretching and mobility exercises. Flexibility exercises should be completed on 2–3 days per week.

Neuromotor Exercise

Neuromotor exercise includes balance, agility, and coordination. Neuromotor exercises should be completed on 2-3 days per week.

Type of Physical Activity

Here are some examples of the different types of exercise!

Cardiorespiratory/Aerobic Exercise

Walking, jogging, swimming, cycling

Strength/Resistance Exercise

Squats, lunges, arms exercises using a light weight, e.g. bottle of water

Flexibility Exercise

Yoga, stretching, joint range of motion exercises

Neuromotor Exercise

Balance exercises, Tai chi
