# Supplementary material for: Promoting physical activity in rheumatoid arthritis through a physiotherapist led behaviour change-based intervention (PIPPRA): a feasibility randomised trial
Source: Rheumatol Int. 2024 Mar 4;44(5):779–93. doi: 10.1007/s00296-024-05544-1 (PMC10980645; doi:10.1007/s00296-024-05544-1)
Supplement: Supplementary file 3 — Supplementary file3 (PDF 131 KB) [file 296_2024_5544_MOESM3_ESM.pdf]

PIPPRA

Intervention detail log sheet

**Date:**<br><br>**Start Time:** **Finish time:**	**Session No:**<br><br>**Delivery Mode:**
**Participant Name:**	**Physiotherapist:**
**Physical activity prescription:**<br><br>**Aerobic** ☐<br>**Strengthening** ☐	 <br>**Flexibility** ☐<br>**Balance/Proprioceptive** ☐
**Physical activity prescription details (Record F.I.T.T. details)**	
**Behaviour Change Techniques details (see BCT Taxonomy)**	
**Adverse event(s)**<br>**Yes** ☐<br>**No** ☐	**Adverse event(s) details**
**Additional information**	
