# Supplementary material for: Promoting physical activity in rheumatoid arthritis through a physiotherapist led behaviour change-based intervention (PIPPRA): a feasibility randomised trial
Source: Rheumatol Int. 2024 Mar 4;44(5):779–93. doi: 10.1007/s00296-024-05544-1 (PMC10980645; doi:10.1007/s00296-024-05544-1)
Supplement: Supplementary file 4 — Supplementary file4 (PDF 335 KB) [file 296_2024_5544_MOESM4_ESM.pdf]

Intervention details log

Participant: ________________________________

			Session Number			
**No.**	**Label**	**Definition**	**1**	**2**	**3**	**4**
**1.1**	***Goal setting (behavior)***	Set or agree on a goal defined in terms of the behavior to be achieved				
**1.2**	***Problem solving***	Analyse , or prompt the person to analyse, factors influencing the behavior and generate or select strategies that include overcoming barriers and/or increasing facilitators (includes '**Relapse Prevention**' *and* '**Coping Planning**')				
**1.3**	***Goal setting (outcome)***	Set or agree on a goal defined in terms of a positive **outcome** of wanted behavior				
**1.4**	***Action planning***	Prompt detailed planning of performance of the behavior (must include at least one of context, frequency, duration and intensity). Context may be environmental (physical or social) or internal (physical, emotional or cognitive) (includes '**Implementation Intentions**')				
**1.5**	***Review behavior goal(s)***	Review behavior goal(s) jointly with the person and consider modifying goal(s) or behavior change strategy in light of achievement. This may lead to re-setting the same goal, a small change in that goal or setting a new goal instead of (or in addition to) the first, or no change				
**1.6**	***Discrepancy between current behavior and goal***	Draw attention to discrepancies between a person's current behavior (in terms of the *form, frequency, duration, or intensity* of that behavior) and the person's previously set outcome goals, behavioral goals or action plans (goes beyond self-monitoring of behavior)				
**1.7**	***Review outcome goal(s)***	Review outcome goal(s) jointly with the person and consider modifying goal(s) in light of achievement. This may lead to re-setting the same goal, a small change in that goal or setting a new goal instead of, or in addition to the first.				
**1.8**	***Behavioral contract***	Create a written specification of the behavior to be performed, agreed on by the person, and witnessed by another				
**1.9**	***Commitment***	Ask the person to affirm or reaffirm statements indicating commitment to change the behavior				

			Session Number			
**No.**	**Label**	**Definition**	**1**	**2**	**3**	**4**
**2.1**	***Monitoring of behavior by others without feedback***	Observe or record behavior with the person's knowledge as part of a behavior change strategy				
**2.2**	***Feedback on behavior***	Monitor and provide informative or evaluative feedback on performance of the behavior *(e.g. form, frequency, duration, intensity)*				
**2.3**	***Self-monitoring of behavior***	Establish a method for the person to monitor and record their behavior(s) as part of a behavior change strategy				
**2.4**	***Self-monitoring of outcome(s) of behavior***	Establish a method for the person to monitor and record the **outcome(s)** of their behavior as part of a behavior change strategy				
**2.5**	***Monitoring outcome(s) of behavior by others without feedback***	Observe or record outcomes of behavior with the person's knowledge as part of a behavior change strategy				
**2.6**	***Biofeedback***	Provide feedback about the body *(e.g. physiological or biochemical state)* using an external monitoring device as part of a behavior change strategy				
**2.7**	***Feedback on outcome(s) of behavior***	Monitor and provide feedback on the outcome of performance of the behavior				
**3.1**	***Social support (unspecified)***	Advise on, arrange or provide social support *(e.g. from friends, relatives, colleagues,' buddies' or staff)* or non-contingent praise or reward for performance of the behavior. It includes encouragement and counselling, but only when it is directed at the **behavior**				
**3.2**	***Social support (practical)***	Advise on, arrange, or provide **practical** help *(e.g. from friends, relatives, colleagues, 'buddies' or staff)* for performance of the behavior				
**3.3**	***Social support (emotional)***	Advise on, arrange, or provide **emotional** social support *(e.g. from friends, relatives, colleagues, 'buddies' or staff)* for performance of the behavior				
**4.1**	***Instruction on how to perform a behavior***	Advise or agree on how to perform the behavior (includes '**Skills training**')				
**4.2**	***Information about antecedents***	Provide information about antecedents				
**4.3**	***Re-attribution***	Elicit perceived causes of behavior and suggest alternative explanations *(e.g. external or internal and stable or unstable)*				

			Session Number			
**No.**	**Label**	**Definition**	**1**	**2**	**3**	**4**
**4.4**	***Behavioral experiments***	Advise on how to identify and test hypotheses about the behavior, its causes and consequences, by collecting and interpreting data				
**5.1**	***Information about health consequences***	Provide information (e.g. written, verbal, visual) about health consequences of performing the behavior				
**5.2**	***Salience of consequences***	Use methods specifically designed to **emphasise** the consequences of performing the behaviour with the aim of making them more memorable (goes beyond informing about consequences)				
**5.3**	***Information about social and environmental consequences***	Provide information (e.g. written, verbal, visual) about social and environmental consequences of performing the behavior				
**5.4**	***Monitoring of emotional consequences***	Prompt assessment of **feelings** after attempts at performing the behavior				
**5.5**	***Anticipated regret***	Induce or raise awareness of expectations of future regret about performance of the unwanted behavior				
**5.6**	***Information about emotional consequences***	Provide information (e.g. written, verbal, visual) about emotional consequences of performing the behavior				
**6.1**	***Demonstration of the behavior***	Provide an observable sample of the performance of the behaviour, directly in person or indirectly e.g. via film, pictures, for the person to aspire to or imitate (includes '**Modelling**').				
**6.2**	***Social comparison***	Draw attention to others' performance to allow comparison with the person's own performance				
**6.3**	***Information about others' approval***	Provide information about what other people think about the behavior. The information clarifies whether others will like, approve or disapprove of what the person is doing or will do				
**7.1**	***Prompts/cues***	Introduce or define environmental or social stimulus with the purpose of prompting or cueing the behavior. The prompt or cue would normally occur at the time or place of performance				
**7.2**	***Cue signalling reward***	Identify an environmental stimulus that reliably predicts that reward will follow the behavior (includes ***'Discriminative cue'***)				
**7.3**	***Reduce prompts/cues***	Withdraw gradually prompts to perform the behavior (includes ***'Fading'***)				
**7.4**	***Remove access to the reward***	Advise or arrange for the person to be separated from situations in which unwanted behavior can be rewarded in order to reduce the behavior (includes ***'Time out'***)				
**7.5**	***Remove aversive stimulus***	Advise or arrange for the removal of an aversive stimulus to facilitate behavior change (includes ***'Escape learning'***)				

			Session Number			
**No.**	**Label**	**Definition**	**1**	**2**	**3**	**4**
**7.6**	***Satiation***	Advise or arrange repeated exposure to a stimulus that reduces or extinguishes a drive for the unwanted behavior				
**7.7**	***Exposure***	Provide systematic confrontation with a feared stimulus to reduce the response to a later encounter				
**7.8**	***Associative learning***	Present a neutral stimulus jointly with a stimulus that already elicits the behavior repeatedly until the neutral stimulus elicits that behavior (includes **'Classical/Pavlovian Conditioning'**)				
**8.1**	***Behavioral practice/ rehearsal***	Prompt practice or rehearsal of the performance of the behavior one or more times in a context or at a time when the performance may not be necessary, in order to increase habit and skill				
**8.2**	***Behavior substitution***	Prompt substitution of the unwanted behavior with a wanted or neutral behavior				
**8.3**	***Habit formation***	Prompt rehearsal and repetition of the behavior in the same context repeatedly so that the context elicits the behavior				
**8.4**	***Habit reversal***	Prompt rehearsal and repetition of an alternative behavior to **replace** an unwanted habitual behavior				
**8.5**	***Overcorrection***	Ask to repeat the wanted behavior in an exaggerated way following an unwanted behaviour				
**8.6**	***Generalisation of a target behavior***	Advise to perform the wanted behaviour, which is already performed in a particular situation, in another situation				
**8.7**	***Graded tasks***	Set easy-to-perform tasks, making them increasingly difficult, but achievable, until behavior is performed				
**9.1**	***Credible source***	Present verbal or visual communication from a credible source **in favour of or against the behavior**				
**9.2**	***Pros and cons***	Advise the person to identify and compare reasons for wanting (pros) and not wanting to (cons) change the behavior (includes '**Decisional balance'**)				
**9.3**	***Comparative imagining of future outcomes***	Prompt or advise the imagining and comparing of future outcomes of changed versus unchanged behaviour				
**10.1**	***Material incentive (behavior)***	Inform that money, vouchers or other valued objects ***will be*** delivered if and only if there has been effort and/or progress in performing the behavior (includes **'Positive reinforcement'**)				
**10.2**	***Material reward (behavior)***	Arrange for the delivery of money, vouchers or other valued objects if and only if there ***has been*** effort and/or progress in performing the behavior (includes '**Positive reinforcement'**)				
**10.3**	***Non-specific reward***	Arrange delivery of a reward if and only if there ***has been*** effort and/or progress in performing the behavior (includes '**Positive reinforcement'**)				
**10.4**	***Social reward***	Arrange verbal or non-verbal reward if and only if there ***has been*** effort and/or progress in performing the behavior (includes '**Positive reinforcement**')				

			Session Number			
**No.**	**Label**	**Definition**	**1**	**2**	**3**	**4**
**10.5**	***Social incentive***	Inform that a verbal or non-verbal reward ***will be*** delivered if and only if there has been effort and/or progress in performing the behavior (includes '**Positive reinforcement'**)				
**10.6**	***Non-specific incentive***	Inform that a reward ***will be*** delivered if and only if there has been effort and/or progress in performing the behavior (includes '**Positive reinforcement'**)				
**10.7**	***Self-incentive***	Plan to reward self in future if and only if there has been effort and/or progress in performing the behavior				
**10.8**	***Incentive (outcome)***	Inform that a reward ***will be*** delivered if and only if there has been effort and/or progress in achieving the behavioural **outcome** (*includes* ***'*****Positive reinforcement*'***)				
**10.9**	***Self-reward***	Prompt self-praise or self-reward if and only if there ***has been*** effort and/or progress in performing the behavior				
**10.10**	***Reward (outcome)***	Arrange for the delivery of a reward if and only if there ***has been*** effort and/or progress in achieving the behavioral **outcome** (includes '**Positive reinforcement**')				
**10.11**	***Future punishment***	Inform that future punishment or removal of reward will be a consequence of performance of an unwanted behavior (may include fear arousal) (includes ***'*****Threat*'***)				
**11.1**	***Pharmacological support***	Provide, or encourage the use of or adherence to, drugs to facilitate behavior change				
**11.2**	***Reduce negative emotions*** [b]	Advise on ways of reducing negative emotions to facilitate performance of the behavior (includes '**Stress Management**')				
**11.3**	***Conserving mental resources***	Advise on ways of minimising demands on mental resources to facilitate behavior change				
**11.4**	***Paradoxical instructions***	Advise to engage in some form of the unwanted behavior with the aim of reducing motivation to engage in that behaviour				
**12.1**	***Restructuring the physical environment***	Change, or advise to change the **physical** environment in order to facilitate performance of the wanted behavior or create barriers to the unwanted behavior (other than prompts/cues, rewards and punishments)				
**12.2**	***Restructuring the social environment***	Change, or advise to change the **social** environment in order to facilitate performance of the wanted behavior or create barriers to the unwanted behavior (other than prompts/cues, rewards and punishments)				

			Session Number			
**No.**	**Label**	**Definition**	**1**	**2**	**3**	**4**
**12.3**	***Avoidance/reducing exposure to cues for the behavior***	Advise on how to avoid exposure to specific social and contextual/physical cues for the behavior, including changing daily or weekly routines				
**12.4**	***Distraction***	Advise or arrange to use an alternative focus for attention to avoid triggers for unwanted behaviour				
**12.5**	***Adding objects to the environment***	Add objects to the environment in order to facilitate performance of the behavior				
**12.6**	***Body changes***	Alter body structure, functioning or support **directly** to facilitate behavior change				
**13.1**	***Identification of self as role model***	Inform that one's own behavior may be an example to others				
**13.2**	***Framing/reframing***	Suggest the deliberate adoption of a perspective or new perspective on behavior (e.g. its purpose) in order to change cognitions or emotions about performing the behavior (includes '**Cognitive structuring**')				
**13.3**	***Incompatible beliefs***	Draw attention to discrepancies between current or past behavior and self-image, in order to create discomfort (includes **'Cognitive dissonance'**)				
**13.4**	***Valued self-identity***	Advise the person to write or complete rating scales about a cherished value or personal strength as a means of affirming the person's identity as part of a behavior change strategy (includes **'Self-affirmation'**)				
**13.5**	***Identity associated with changed behavior***	Advise the person to construct a new self-identity as someone who 'used to engage with the unwanted behavior'				
**14.1**	***Behavior cost***	Arrange for withdrawal of something valued if and only if an unwanted behavior is performed (includes '**Response cost'**).				
**14.2**	***Punishment***	Arrange for aversive consequence contingent on the performance of the unwanted behavior				
**14.3**	***Remove reward***	Arrange for discontinuation of contingent reward following performance of the unwanted behavior (includes **'Extinction'**)				
**14.4**	***Reward approximation***	Arrange for reward following any approximation to the target behavior, gradually rewarding only performance closer to the wanted behavior (includes **'Shaping'**)				
**14.5**	***Rewarding completion***	Build up behavior by arranging reward following final component of the behavior; gradually add the components of the behavior that occur earlier in the behavioral sequence (includes **'Backward chaining'**)				

			Session Number			
**No.**	Label	**Definition**	**1**	**2**	**3**	**4**
**14.6**	***Situation-specific reward***	Arrange for reward following the behavior in one situation but not in another (includes ***'Discrimination training'***)				
**14.7**	***Reward incompatible behavior***	Arrange reward for responding in a manner that is incompatible with a previous response to that situation (includes **'Counter-conditioning'**)				
**14.8**	***Reward alternative behavior***	Arrange reward for performance of an alternative to the unwanted behavior (includes ***'Differential reinforcement'***)				
**14.9**	***Reduce reward frequency***	Arrange for rewards to be made contingent on increasing duration or frequency of the behavior (includes ***'Thinning'***)				
**14.10**	***Remove punishment***	Arrange for removal of an unpleasant consequence contingent on performance of the wanted behavior (includes **'Negative reinforcement')**				
**15.1**	***Verbal persuasion about capability***	Tell the person that they can successfully perform the wanted behavior, arguing against self-doubts and asserting that they can and will succeed				
**15.2**	***Mental rehearsal of successful performance***	Advise to practise imagining performing the behavior successfully in relevant contexts				
**15.3**	***Focus on past success***	Advise to think about or list previous successes in performing the behavior (or parts of it)				
**15.4**	***Self-talk***	Prompt positive self-talk (aloud or silently) before and during the behavior				
**16.1**	***Imaginary punishment***	Advise to imagine performing the **unwanted** behavior in a real-life situation followed by imagining an unpleasant consequence (includes ***'Covert sensitisation'***)				
**16.2**	***Imaginary reward***	Advise to imagine performing the **wanted** behavior in a real-life situation followed by imagining a pleasant consequence (includes ***'Covert conditioning'***)				
**16.3**	***Vicarious consequences***	Prompt observation of the consequences (including rewards and punishments) for others when they perform the behavior				
