# Supplementary material for: Promoting physical activity in rheumatoid arthritis through a physiotherapist led behaviour change-based intervention (PIPPRA): a feasibility randomised trial
Source: Rheumatol Int. 2024 Mar 4;44(5):779–93. doi: 10.1007/s00296-024-05544-1 (PMC10980645; doi:10.1007/s00296-024-05544-1)
Supplement: Supplementary file 5 — Supplementary file5 (PDF 144 KB) [file 296_2024_5544_MOESM5_ESM.pdf]

Ethics Appendix 7: Interview Guides

The interview guides below are for four distinct groups who are involved in the PIPPRA pilot study.

Group a) Intervention group interview question guide

- What was your experience of the intervention?
- Are there any aspects of the content or delivery of intervention you are unhappy with?
- Were all aspects of the recruitment process acceptable to you?
- How did you find the process of giving informed consent?
- What was your experience of the various outcome measures?
- How was the delivery of the physical activity component i.e. what are your views on the frequency, intensity, time & type of the intervention?
- How was your experience of the delivery of the behaviour change component,
- Was the setting for delivery of the pilot intervention acceptable?
- Could the support resources for the pilot intervention be improved?
- Were there any unintended consequences from the intervention?

Group b) Comparator group interview questions

- How did you find being in the control group?
- Was the physical activity leaflet useful? Was it easy to read, informative? Are there any changes to be made to it?
- Were all aspects of the recruitment process acceptable to you?
- How did you find the process of giving informed consent?
- What was your experience of the various outcome measures?

Group c) Deliverer of the intervention interview questions

- Does the planned intervention need to be refined/adapted to make it more acceptable to participants, to other physiotherapists who may not have a background in rheumatology and behaviour change and more useful in the setting it was delivered in?
- How might the intervention have to be adapted to be suitable on a full trial?
- Are participants unhappy/uncomfortable with any aspects of the intervention?
- Is the intervention the right amount of treatment?
- How well prepare to deliver the intervention did you feel?

Group d) Clinic staff supporting recruitment

- Have you any suggestions on how to make recruiting participants easier in a busy clinic environment?
- When do you think is the best way to give the study information to potential participants?
- Where is the best place to do that? Is there a location that is best?
- Do potential participants ask you any questions about the study?
